# Towards Understanding the Function of Aegerolysins

**DOI:** 10.3390/toxins14090629

**Published:** 2022-09-11

**Authors:** Nada Kraševec, Matej Skočaj

**Affiliations:** 1 Department of Molecular Biology and Nanobiotechnology, National Institute of Chemistry, SI-1000 Ljubljana, Slovenia; 2 Department of Biology, Biotechnical Faculty, University of Ljubljana, SI-1000 Ljubljana, Slovenia

**Keywords:** aegerolysins, AlphaFold structure modeling, bacteria, competitive exclusion, fungi, insecticidal, lipid binding, lifestyle, membrane-attack complex/perforin domain (MACPF), pore forming proteins

## Abstract

Aegerolysins are remarkable proteins. They are distributed over the tree of life, being relatively widespread in bacteria and fungi, but also present in some insects, plants, protozoa, and viruses. Despite their abundance in cells of certain developmental stages and their presence in secretomes, only a few aegerolysins have been studied in detail. Their function, in particular, is intriguing. Here, we summarize previously published findings on the distribution, molecular interactions, and function of these versatile aegerolysins. They have very diverse protein sequences but a common fold. The machine learning approach of the AlphaFold2 algorithm, which incorporates physical and biological knowledge of protein structures and multisequence alignments, provides us new insights into the aegerolysins and their pore-forming partners, complemented by additional genomic support. We hypothesize that aegerolysins are involved in the mechanisms of competitive exclusion in the niche.

## 1. Introduction

The aegerolysin family (Pfam 06355) is a lesser-known protein family that has received increasing attention in recent years. The aegerolysin family consists of proteins that are biochemically characterized as β-structured proteins and share some common features: similar small molecular weights (15–20 kDa), low isoelectric points, and stability in a wide pH range [1]. Because they are non-core proteins, without a member of this protein family in each of the sequenced fungi, their distribution among fungal species is inconsistent, and different numbers of homologs have been reported for species within the same genus [2,3,4]. They are not only relatively widespread in fungi and bacteria, but also identified in few plants, protozoa, viruses, and insects [1,2]. 

In recent years, several reviews of this protein family have been published, but none of them included data on the ecology of the organisms producing them. In particular, their function is enigmatic, although some authors suggest a role in the development of the organism [1,2,3]. However, some of them function as two-component cytolysins that exhibit membrane permeabilization activity together with another non-aegerolysin-like protein [2,5]; these act together to perforate natural and artificial lipid membranes [1,2,5]. The aegerolysin-like proteins provide membrane lipid selectivity and recruit partner protein molecules to form a pore complex inserted into the membrane [2,5]. 

Despite the limited scientific knowledge about the function of aegerolysins, several potential applications are already emerging. Most commonly, some fungal aegerolysins serve as probes for the detection, labeling, and imaging of specific membrane lipids, lipid rafts, cancer cells, invertebrates, or parasites [4,6,7,8,9,10,11]. In high concentrations, they can induce both artificial lipid vesicles as well as live cells, such as blood cells or neuroblastoma cells, to bend and bud [10]. A role of some aegerolysins in combating obesity and related metabolic disorders has been recognized [10]. Their genes and expression may serve as markers for the progression of fruiting body differentiation during mushrooms cultivation [10] or as biomarkers to detect fungal exposure and progression of infectious disease [3,4]. In addition, antibodies produced against aegerolysins can serve as immuno-diagnostic tools [4]. Due to their variable sequence, aegerolysins serve as tools to identify of fungal phytopathogen isolates compared to some closely related species where the internal transcribed spacer barcoding method has failed [4]. Strong promoters regulating aegerolysin genes can promote the secretion of heterologous proteins from fungi in concomitant multi-gene expression [4]. Certain aegerolysins that combine with larger protein partners to form pore-forming complexes can be used to selectively eliminate insect pests [4,10] or to treat certain types of cancer cells [4,10]. 

Currently, a total of 2303 sequenced genomes of fungal species are deposited in the largest public database of fungal genomes [12]. Aegerolysins are encoded in the genomes of Dikarya fungi but are absent in some clades, e.g., Saccharomycotina [4]. Fungal genomes contain 949 protein sequences with the recognized aegerolysin domain PF06355 [12]. In 31,332 genomes in a bacterial database, there were 169 hits for aegerolysins [13]. Despite their presence in cells at certain developmental stages and their presence in secretomes, few aegerolysins have been studied in detail.

Here we provide a complete overview of the lifestyles of organisms (fungi, bacteria, insects, and viruses) in which aegerolsyins have been identified not only by genome sequencing, but also with at least some previously described functional properties. Their putative structures were predicted using the recently introduced successful prediction tool AlphaFold2 to further improve the knowledge of their function [14]. The presence of putative bicomponent partner proteins or additional aegerolysin gene copies was also investigated. 

## 2. Aegerolysins

We have collected (experimental) published data on 23 different aegerolysins and their variants. In total, they were characterized from 18 different species belonging to different kingdoms of tree of life. Twelve of these aegerolysins belong to fungi, four to bacteria, and one to insects and viruses. In fungi, they were characterized from four mushrooms (*Agaricomycotina*) from the order *Agaricales*: *Pleurotus ostreatus*, *P. eryngii*, *Agrocybe aegerita*, and *Moniliophthora pernicious*, as well as in the ordo *Polyporales*: *Lignosus rhinocerotis* (Table 1). The origin of these aegerolysins were also four filamentous *Eurotimycetes* from the ordo *Eurotiales*: *Aspergillus fumigatus*, *A. niger*, *A. terreus*, and *A. oryzae* (Table 1). Two species belonged to the *Sordariomycetes*, ordo *Hypocreales*: *Beauveria bassiana*, and *Trichoderma atroviride*, and another to the *Dothideomycetes*, ordo *Pleosporales*: *Alternaria geisen* (Table 1). There were four bacterial species, two belonging to *Firmicutes*: *Bacillus thuringiensis* and *Clostridium bifermentans*, and another two to *Proteobacteria*: *Pseudomonas aeruginosa* and *Alcaligenes faecalis* (Table 1). Another species belongs to *Insecta—Lepidoptera*, *Noctuidae*: *Pseudoplusia includes*, and another to *Varidnaviria*, *Ascoviridae*: *Trichoplusia ni ascovirus* 2c (Table 1).

### 2.1. Fungal Aegerolysins from Basidiomycota—Agaricomycotina 

#### 2.1.1. Aegerolysins from *Pleurotus ostreatus*

The oyster mushroom or hiratake, *Pleurotus ostreatus* (Jacq. ex Fr.) P. Kumm. (1871) (*Agaricales*), is widely distributed in many temperate and subtropical forests. As a saprotroph, a white-rot wood fungus, it is a primary decomposer of wood, especially deciduous trees such as beech. It is also known to paralyze nematodes and consume prey [15]. As a common edible mushroom, it is grown commercially for food throughout the world.

Pleurotolysin A (**PlyA**, **PriA**), ostreolysin (**OlyA**), ostreolysin A6 (**OlyA6**), recombinant ostreolysin (**rOlyA**), are aegerolysins from the mushroom *P. ostreatus.* According to genome sequences of *P. ostreatus* strains PC9 and PC15, they are encoded by the same locus [52,53,54,55,56]. 

**Figure 1:**
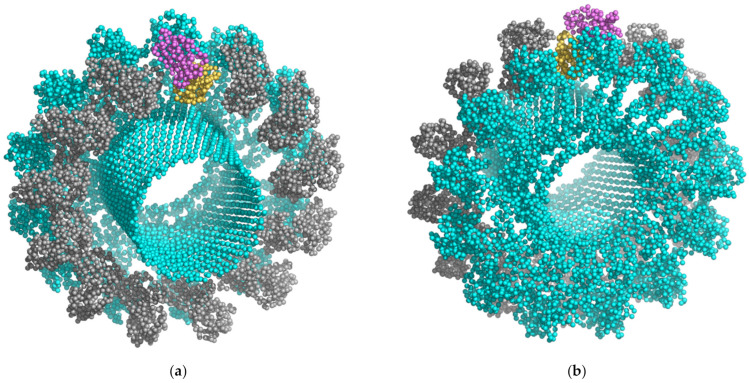
Pore-forming pleurotolysin complex of *Pleurotus ostreatus*. The hetero 39-mer structure of the pore was solved (PDB ID: 4V2T) [60]; it consists of 26 aegerolysin molecules PlyA and 13 MACPF protein molecules PlyB; the size of the pore is 8 × 10 nm. (**a**) Bottom view; (**b**) Top view; PlyA, pleurotolysin A—in grey, magenta, and orange; PlyB, pleurotolysin B—in cian. Presentation of the structure by PyMOL [62].

In 1979, it was discovered that *P. ostreatus* encodes the hemolytic protein pleurotolysin with a size of 15 kDa, and these first experiments showed the importance of sphingomyelin (SM) for **Ply** binding [57]. A clone containing aegerolysin gene was the most abundant expressed sequence tag (EST) in the primordial and the fruit body library [58]. Then, 25 years later, in 2004, it was revealed that this **Ply** protein is actually composed of subunits A and B [59]. Component A (**PlyA**), a 15 kDa aegerolysin, binds to SM/cholesterol (Chol)-rich membranes and then recruits a 59 kDa membrane attack complex/perforin (MACPF)-domain containing protein partner, **PlyB**, which then leads to transmembrane pore formation and cell lysis [59]. The combination of X-ray diffraction and cryo-electron microscopy has shown that **PlyA** paired with **PlyB** forms a 39-heteromeric membrane-embedded pore with a 13-fold symmetry structure (PDB ID: 4V2T) (Figure 1) [60]. **PlyA** has a β-sandwich structure similar to the actinoporin family (PF06369) of pore-forming proteins [60]. For adequate binding of **PlyA**, the Chol content should be more than 30 mol% [59]. **PlyA**/**PlyB** nanopores have been recently proposed as sequencing tools [61].

In 2002, another group of researchers isolated a very similar 15 kDa protein from the same mushroom and named it ostreolysin (**Oly**) [63]. The primary structure of **Oly** is 79% (29 amino acids substitution) identical to **PlyA** [11]. The first experiments with the native isolate of **Oly** showed that it is cytolytic for erythrocytes and various cell lines [64,65]. Since the expression of **Oly** occurs during the formation of primordia and fruiting bodies of the mushroom, it has been speculated that it plays a role in the fruiting bodies of the mushroom [63,66,67]. Biochemical experiments in 2004 showed that **Oly** also specifically recognizes membranes enriched in SM and Chol [68,69], and that binding of a protein is abolished after pretreatment of cells with methyl-β-cyclodextrin or lysophospholipids [70,71]. However, as in the case of **Ply**, it was later revised that native isolate of **Oly** (**OlyA**) was contaminated with **PlyB**, which was responsible for the observed cytolytic effects [72]. 

Ostreolysin A6 (**OlyA6**), which has 78% identity (31 amino acids substitution) with **OlyA** [11], was expressed as recombinant protein in 2013 [72]. First experiments showed that **OlyA6** specifically bind to SM/Chol-rich lipid membranes and has very similar binding properties to **PlyA** [72]. Recently, **OlyA6** was confirmed to bind to SM/Chol lipid complexes and not to free SM (PDB ID: 6MYJ) [73]. **OlyA6** was later fused to the fluorescent protein mCherry, and was used as a lipid raft marker [74]. **OlyA6**-mCherry was shown to be non-toxic to vertebrate cells and, due to its stability and small size, it became an important tool for studying lipid rafts [74]. **OlyA6**-mCherry stained lipid rafts in plasma membranes in live and fixed Madin-Darby canine kidney (MDCK) cells did not colocalize with other raft markers, that sense individual raft-residing lipids, and did not bind to intracellular membranes, and its binding was abolished by methyl-β-cyclodextrin or sphingomyelinase pretreatment [74]. However, it has been observed that excessive concentrations of **OlyA6** or **Oly6**-mCherry can lead to membrane vesiculation [72,74,75]. In addition, **OlyA6**-mCherry applied to live MDCK cells was internalized via caveolin-dependent pathway and was detected near the Golgi complex after 90 min [74]. Similar to the combination of **PlyA** and **PlyB** or **OlyA** and **PlyB**, the mixtures of **OlyA6** and **PlyB** form transmembrane pores, leading to cell lysis [72]. The combination of lipid raft sensing **OlyA6** and **PlyB** can be used as anticancer treatment for cancers with increased concentration of lipid rafts in their membranes as is the case in urothelial cancer cells [76]. Recently, it was revealed that **OlyA6** has another high affinity lipid receptor, ceramide phosphoethanolamine (CPE), which is specific for cell membranes of invertebrates and some bacteria [77,78,79]. **OlyA6** binds 1000-fold more strongly to vesicles composed of CPE and Chol than to vesicles composed of SM/Chol [80], and it also binds to membranes with physiological concentrations (5 mol%) of CPE [78,79]. In addition to labelling lipid rafts, **OlyA6** can also be used as a marker to label CPE in insect cells and tissues [79,80,81]. Furthermore, **OlyA6**/**PlyB** protein mixtures permeabilize lipid vesicles containing ≤5 mol% CPE and are toxic to CPE-containing Sf9 cells, as well as to the larvae of Colorado potato beetle (*Leptinotarsa decemlineata* Say, 1824) and western corn rootworm (*Diabrotica virgifera virgifera* LeConte, 1868), and thus have been proposed as new environmentally friendly biopesticides [10,79] with low toxicity for non-target organisms [82] and no toxicity for humans after ingestion due to their fast decomposition by mammalian digestive enzymes [83]. It must be noted, however, that the intravenous application of the native **OlyA6**/**PlyB** isolate in rodents resulted in a cardiorespiratory arrest and can induce severe histopathological changes in kidneys, lungs, and heart [84,85]. The half lethal dose (LD50) in mice is 1.17 mg/kg [86]. Very recently, another lipid receptor of **OlyA6**, ceramide aminoethylphosphonate (CAEP), a CPE analogue of marine invertebrates such as mollusks and cnidarians, was identified [87]. Moreover, **OlyA6**/**PlyB** complexes triggered strong lysis of CAEP–lipid containing vesicles. 

In 2017, another aegerolysin from *P. ostreatus* was produced in recombinant form and named recombinant ostreolysin (**rOlyA**) [88], which has an amino acid substitution at position 50 (valine to isoleucine) compared to OlyA6. **rOlyA** has not been used as a lipid raft marker, but it has been shown to have anti-proliferative and pro-apoptotic effects against colon cancer cells or can be used the treatment of metabolic disorders, which was attributed to its lipid raft binding properties [88]. **rOlyA** induced apoptosis of human and mouse cancer cells in vitro but was not toxic to normal intestinal cells [88]. After endocytosis, **rOlyA** colocalized with β-III tubulin, which probably impaired microtubule dynamics and led to cell death. These results were confirmed by in vivo mouse models in which **rOlyA** reduced tumor cell growth. Further, through its lipid raft binding and interfering with signaling cascades, **rOlyA** can induce browning of white preadipocytes used for energy storage in vitro, resulting in a brown-like phenotype with increased mitochondrial biogenesis. **rOlyA** has therefore been proposed as a candidate for regulating metabolic disorders such as obesity, hyperlipidemia, and non-alcoholic fatty liver disease [89,90].

In the genomes of *P. ostreatus* PC9 or PC15, a bi-directional pair of genes with 5′–5′ orientation of the **plyA** (**priA**) and **plyB** genes was observed [91,92]; no difference among the two strains was observed in the sequences of PlyA.

#### 2.1.2. Aegerolysins from *Pleurotus eryngii*

Boletus of the steppes *Pleurotus eryngii* (DC.) Quél. 1872 (*Agaricales*), commonly called king oyster or trumpet or brown mushroom, French horn mushroom, or Aliʻi oyster, is an edible mushroom. It is of commercial interest because of the mild taste and pleasant odor of its fruiting bodies. In addition, its mycelium is often grown on lignocellulosic wastes (such as wheat straw). Unlike *P. ostreatus*, this fungus lives saprobically or facultatively biotrophic on roots of herbaceous plants of the *Apiaceae* family as a grassland-litter decomposer. *Pleurotus eryngii sensu stricto* consists of at least five varieties such as var. *eryngii*, *ferulae*, *elaeoselini*, *thapsiae*, and *tingitanus*, which differ significantly in their habitat distribution, host, and morphology [16]. It possesses nematocidal properties as well as *P. ostreatus* [15].

Pleurotolysin A2 (**PlyA2**) and erylysin (**EryA**) are two aegerolysins from *P. eryngii*. In 2006, a 17-kDa hemolysin was isolated from fruiting bodies of the mushroom *P. eryngii* and was originally named eryngeolysin [93]. Its N-terminal sequence showed high homology to **OlyA** from *P. ostreatus* and aegerolysin from *Agrocybe aegerita*. Its hemolytic activity remained unchanged over the pH range of 4 to 12. It also showed antibacterial activity against *Bacillus megaterium* and *B. subtilis*, but not against some other bacteria such as *Staphylococcus aureus*, *Escherichia coli*, *Enterobacter aerogenes*, *Pseudomonas aeruginosa*, *P. fluorescens*, *Mycobacterium phlei*, and *Proteus vulgaris*, or fungal species *Botrytis cinerea*, *Fusarium oxysporum*, *Mycosphaerella arachidicola*, *Physalospora piricola*, and *Rhizoctonia solani* [93]. The sensitivity of mammalian erythrocytes to eryngeolysin was the highest, followed by birds, reptiles, and fish erythrocytes [93]. It also showed cytotoxicity against leukemia cells (L1210), and inhibited mitogenic responses of mouse splenocytes. N-glycolyneuraminic acid was the only sugar that could inhibit hemolytic activity [93]. 

In 2013, the sedimentation assay of the crude extract of *P. eryngii* revealed that pleurotolysin A2 (**PlyA2**) is also a SM/Chol-binding protein [94]. **PlyA2** has two amino acid substitutions compared to **PlyA** [95], while it has 93% sequence identity (nine substitutions) with **OlyA6** [94]. Since the recombinant protein showed no hemolytic activity or toxicity to Hela cells it was then prepared as recombinant fluorescently labeled protein, **PlyA2**-EGFP, which was used as a lipid raft marker. **PlyA2** successfully labeled Hela cells, and binding of the protein was abolished after the pretreatment of the cells with methyl-β-cyclodextrin or sphingomyelinase. **PlyA2**-EGEP showed substantial colocalization with the raft-associated protein CD59. After permeabilization of the cells, **PlyA2** was found to bind to late endosomes and, similar to **OlyA6**, not to cytoplasm-facing membranes. As with **OlyA6**, **PlyA2** was later shown to bind more strongly to CPE/Chol-containing membranes [80]. Interestingly, **PlyA2** was also able to recognize and bind CPE membranes in the absence of Chol, as the only of the tested *Pleurotus* aegerolysins [80]. **PlyA2**-EGFP successfully labeled CPE in *Drosophila* embryonic Kc167 cells and in larvae of the same insects [80]. It is suggested that **PlyA2**/**PlyB** complexes can be used as new bioinsecticides, as has been discussed for **OlyA6**/**PlyB** complexes [79]. 

A novel bicomponent toxin pair was isolated and characterized from *P. eryngii*, **EryA** and **EryB** in 2010 [96]. Most *Pleurotus* aegerolysins (**OlyA**, **OlyA6**, **PlyA**, and **PlyA2**) bind to SM/Chol-rich lipid membranes [5,59,65,74,94,97], but not **EryA**, ortholog of **PlyA2** from *P. eriyngii* [80]. For this reason **EryA**-mCherry was very recently used as a molecular tool for detection of periodontal disease which is in majority of the cases associated with CPE-producing periodontal bacteria [98]. **EryA**-EGFP was also successfully used to label the bloodstream form of the protozoan *Trypanosoma cruzi*, a causative of sleeping disease, that has CPE in its membranes [80]. Recently, **EryA**-EGFP was reported to bind artificial lipid membranes composed of cholesterol, fully saturated phosphatidylcholine and cardiolipin (CL) in a 5:4:1 molar ratio [99]. Furthermore, intracellularly expressed **EryA**-EGFP localized to regions of negative curvature (inner leaflets at cell poles and the outer leaflets at division sites) in *E. coli*, and inhibited cytokinesis [99]. This localization was assigned to **EryA** interaction with CL, a membrane lipid that localizes in curved membranes such as mitochondrial cristae, cell poles and division sites of rod-shaped bacteria.

The genome of *P. eryngii* ATCC 90797, similar to that of *P. ostreatus*, encodes a MACPF-containing protein, erylysin B (**EryB**) [96], which is 97% identical to **PlyB** [10]; it is a neighboring gene of **PlyA2**, whereas **EryA** has no MACPF-like protein neighboring gene. However, it is surprising that some aegerolysins perform better in terms of their cytolytic activity when combined with their non-native partner. This has been studied for **PlyA2** and **EryA**; **PlyA2** show better hemolytic/lipid vesicle permeable activity when combined with the MACP partner **PlyB** from *P. ostreatus*, rather than with their native protein partner, **EryB** [78].

#### 2.1.3. Aegerolysins from *Agrocybe aegerita*

The poplar mushroom *Cyclocybe aegerita* Vizzini 2014 (*Agrocybe aegerita*, *A. cylindracea*, or *Pholiota aegerit*) (*Agaricales*), also known as tea tree mushroom, Cha shu gu, Yanagi-matsutake, velvet pioppini, or sword-belt mushroom, is a high-value commercially grown edible mushroom. It is a saprotrophic, wood-inhabiting fungus that decomposes mainly dead wood of deciduous trees, especially poplar and willow, and causes weak white rot on deciduous trees in temperate forests; it may become pathogenic on some other tree species [17].

The aegerolysin protein family is named after aegerolysin from the mushroom *A. aegerita*) [63]. Northern hybridizations with total RNA extracted from four developmental stages of *A. aegerita* SM51 (*A1B1*/*A2B2*) showed that the **Aa-pri1** gene is specifically expressed during primordia or the immature fruiting body stage and that it is not expressed (at list not to a detectable level) during vegetative growth or late stages of fruiting body maturation [100]. 

**Aa-pri1** is one of six genes for putative aegerolysins encoded in the *A. aegerita* AAE3 genome. Only one out of these genes has a neighboring gene for component B, which is homologous to the MACPF domain-containing protein **PlyB**.

#### 2.1.4. Aegerolysins from *Moniliophthora perniciosa*

The species complex *Moniliophthora perniciosa* (Stahel) Aime and Phillip-Mora (2005) (*Crinipellis perniciosa*) (*Agaricales*) consists of a number of geographically separated populations that infect a broad range of different hosts: biotype C on *Theobroma* and *Herrania* spp. (*Malvaceae*), H on *Heteropterys acutifola* (*Malpighiaceae*), L *Arrabidaea* spp. (*Bignoniaceae*), and S on *Solanum* spp. (*Solanaceae*) [18]. It is most known as a hemibiotrophic fungus that causes witches’ broom disease in cacao plant (*Theobroma cacao* L.) with two distinct growth phases [18].

The ability to culture a biotrophic-like phase of *M. perniciosa* in vitro, as well as the recent findings from the nearly complete genome and expression studies, clearly indicate that these different growth phases of the fungus function under different metabolic parameters [18]. The gene expression profile determined by microarray suggests physiological changes in the mycelia prior to basidiomata formation, and confirmed by RT-qPCR at different stages during cultivation in basidiomata-inducing medium [101]. Of the three putative genes involved in fructification, two are related to the identified **PriA** from *P. ostreatus* and **AA-Pri1** from *A. aerogerita*, respectively, and one is related to **PlyB** from *P. ostreatus*. The expression of aegerolysin **MpPRIA1** was low in yellow and reddish-pink mycelial stages and before stress, but increased about fourfold in mycelia with primordia and 90-fold in basidiomata compared to the initial white mycelial stage [101]. Expression of the aegerolysin **MpPRIA2** increased 17-fold at the reddish-pink mycelium stage, but decreased 11-fold before stress, 4-fold in stressed mycelia, and 47-fold in mycelia with primordia. Transcripts of the gene **MpPRIA2** increased 23-fold in basidiomata, which was lower in mycelia with primordia [101]. Transcripts of the gene **MpPLYB** increased 1.4-fold in the yellow mycelium stage, 15-fold in reddish-pink mycelia, and remained at high levels in mycelia before stress, under stress, and in mycelia with primordia (about 10–12-fold increase), but decreased in basidiomata, where they were only 1.6-fold higher than in white mycelia [101]. **MpPRIA1** and **MpPRIA2** have homologous regions but appear to correspond to two individual genes whose expression is consistent with the morphological differentiation of primary hyphal nodules from primordia. These aegerolysins may contribute to the process of hyphal aggregation, as their expression, albeit at low levels, occurred prior to the appearance of primordia, when hyphae became spherical for primordia formation [102]. This stage coincides with the stage of reddish-pink mycelium, where hyphal nodules are detectable. The exact function of these proteins remains unclear, but their involvement in programmed cell death seems rather unlikely, since aegerolysins from *Pleurotus* have a lytic function and act in SM/Chol membranes at pH between 7 and 8, which is not normally the case in fungal cells [65,103,104]. However, they can also act lytically on membranes composed of SM and ergosterol, although the binding and permeabilization is twice less efficient as for the SM/Chol membranes [69]. 

Six aegerolysins are encoded in the *M. perniciosa* genome according to Pf06355 search. The contigs are too short to detect whether the only homolog of **PlyB—MpPLYB** gene is a neighboring gene of **MpPRIA1** and **MpPRIA2** genes.

#### 2.1.5. Aegerolysins from *Lignosus rhinocerotis*

The tiger milk mushroom *Lignosus rhinocerotis* (Cooke) Ryvarden (1972) (*Polyporales*) is a white rot fungus that develops from the underground tuberous sclerotium. In recent years, this fungus has received much attention because of its extensive wide-range ethnobotanical use as a folk medicine and because of the successful domestication of this once very rare and expensive fungus [19]. 

Systematic profiling of *L. rhinocerotis* sclerotial proteins was performed using 2DE coupled with MALDI-MS and LC-MS. After genome-based proteomic analysis, the aegerolysin domain-containing protein **GME7309** was identified in one of the 45 spots [105].

The genome of *L. rhinocerotis* genome has been published but is not publicly available for browsing [106], so no data on bicomponent partner genes are available.

### 2.2. Fungal Aegerolysins from Ascomycota—Eurotimycetes

The fungal genus *Aspergillus* is of great industrial importance to humans, primarily as a unique cell factory with an exceptionally high secretion capacity for enzymes, but also as a source of important pathogens for humans, animals, and crops, potent carcinogenic food contaminants, and an important genetic model [20]. The genus *Aspergillus* is characterized by high adaptability to different ecological environments. However, the biodiversity of *Aspergillus* in different ecological habitats should always be evaluated in the context of the continuous development of the taxonomy of the genus; the description of new species may reveal a greater biodiversity of the genus and refine the results of previous studies [21].

#### 2.2.1. Aegerolysins from *Aspergillus fumigatus*

*Neosartorya fumigata* O’Gorman, Fuller, and Dyer, 2008 (*Aspergillus fumigatus* Fresenius 1863) (*Eurotiales*), is a ubiquitous saprotrophic fungus that plays an important role in recycling carbon and nitrogen on Earth, but can also be a deadly opportunistic primary and opportunistic pathogen [22]. It is a trimorphic fungus with vegetative mycelium that contributes to the decomposition of soil organic matter, asexual conidia responsible for the aerial dispersal of the species, and dormant ascospores that ensure the long-term survival of the organism. Its natural ecological niche is the soil, where it survives and grows on organic debris. Although this fungal species is not the most abundant in the world, it is one of the most widespread fungal species with airborne conidia. Similar variations were found outdoors and in hospital, with consistently lower numbers indoor. Plant debris in the form of compost piles and stacks of high-moisture hay and straw bales that are self-heating, produce large numbers of spores that can be released into the air, resulting in high but localized numbers when disturbed. The widespread dispersal of decaying leaves after leaf fall is a potential source of smaller spore concentrations, but these are dispersed over a much larger area [23].

In 1962, the first aegerolysin protein, Asp-hemolysin (**Asp-HS**) was described [107], and in 1975 it was isolated from the filamentous fungus *A. fumigatus* [108]. It was not until 1982 that **Asp-HS** was reported to be hemolytic and involved in the pathogenesis of *A. fumigatus* [109]. The nucleotide sequence of a cDNA encoding ***hlyA*** was elucidated in 1994 [110]. **Asp-HS** has been shown to bind specifically to oxidized low-density lipoproteins [111], to have cytotoxic effects on murine macrophages and vascular endothelial cells and to induce cytokine genes [112,113]. However, wild-type and knock-out ***hlyA*** and ***hlyA-like*** mutant strains of *A. fumigatus* have shown that **Asp-HS** production is not important for the progression of invasive alveolar aspergillosis in mice [114]. Instead, **Asp-HS** is thought to play a role in potentiating other virulence mechanisms of the fungus [115]. 

Of the two aegerolysins described, **asp-HS** also has a neighboring partner protein gene **asp-HSB**, and **asp-HS-like** does not in strain *A. fumigatus* Af293 [92]. **asp-HS-like** sequence is also identified as invariant in five other strains, GCA_002234955.1, GCA_002234985.1, A1163, Z5, and var. RP-2014, while for **asp-HS**, variation in one amino acid was observed, and it was absent in var. RP-2014.

#### 2.2.2. Aegerolysins from *Aspergillus niger*

*Aspergillus niger* van Tieghem 1867 (*Eurotiales*) is a filamentous fungus that grows aerobically on organic matter. It is ubiquitous in soil and is commonly reported from indoor environments. *A. niger* is capable of growing in a wide temperature range of 6–47 °C, with a relatively high temperature optimum at 35–37 °C, and it is capable of growing in an extremely wide pH range: 1.4–9.8 [24]. These capabilities and the abundant production of airborne conidiospores result in a ubiquitous occurrence of the species, with a higher incidence in warm and humid locations. Although the main source of Black aspergilli is soil and litter, in compost they are among the most common fungi causing food spoilage and biological decay of other materials. *Aspergillus niger* species complex is often responsible for the postharvest spoilage of fresh fruits (apples, pears, peaches, citrus fruits, grapes, figs, strawberries, tomatoes, melons, etc.) and some vegetables (especially onions, garlic, and sweet potatoes); they are also among the most common fungi isolated from dried fruits, beans, oilseeds, and nuts (peanuts, pecans, pistachios, hazelnuts, almonds, walnuts, etc.) [25,26]. Because *A. niger* has been in use already for many decades to produce extracellular (food) enzymes and citric acid, many of these product are considered generally recognized as safe (GRAS) by the United States Food and Drug Administration [24,27].

Functional studies of the expression of the genes encoding the aegerolysins **NigA1** and **NigA2** and the MACPF-like proteins **NigB1** and **NigB2** suggest that the sporulation process is critical for the strong induction of the expression of all these genes. However, in laboratory environment, deletion of any of the aegerolysin genes had no effect on the growth, development, sporulation efficiency, and phenotype of the mutants, suggesting that aegerolysins are not key factors in the sporulation process [91]. In all expression studies, a strong correlation was observed between the expression of **NigA2** and **NigB1** genes [91]. In *Aspergillus* species, aegerolysins were frequently found as secreted proteins. Their secretion was studied by secretion prediction and Western blotting, and it was confirmed that **NigA1** and **NigA2** are secreted by the fungus [91,92]. Both nigerolysins A are leaderless proteins that reach the cell exterior by unconventional protein secretion [91,92]. The subcellular localization of nigerolysins A in *A. niger* was investigated by immunocytochemistry and live cell imaging. The **NigA** proteins are uniformly distributed in the cytoplasm of fungal hyphae [92]. **NigA2** was shown to interact specifically with an insect-specific membrane sphingolipid, CPE in combination with Chol, but not with SM/Chol [91]. Membranes of insect cells containing this specific sphingolipid were stained after binding the **NigA2** protein labeled with mCherry [91]. Detailed bioinformatics analysis of *Aspergillus* aegerolysins suggests that the same function (target) likely occurs in only a limited number of aegerolysins. From the alignment, chromosomal loci analysis, orthology, synteny, and phylogeny, it appears that the same or similar function described for pairs of pesticidal proteins of *Pleurotus* sp. also occurs in species of the subgenus Circumdati, section Nigri, series Nigri, and several other species with adjacent pairs of putative pesticidal proteins [92]. The combined results suggest that aegerolysins in *A. niger*, and probably in other aspergilli, may be involved in defense against predators.

Of the two aegerolysins described in *A. niger* strain CBS 513.88, **NigA2** also has a neighboring partner protein gene **NigB1**, whereas **NigA1** does not [92]. No variations in aegerolysin sequences for **NigA1** are observed in any of the nine other *A. niger* strains examined: (lacticoffeatus) CBS 101883, (phoenicis Corda) Thom ATCC 13157, ATCC 1015, ATCC van Tieghem 13496, NRRL3, ASM285, ATCC_64974_N402, ASM221148, except for one amino acid variance in ASM151534. The same is also observed for **NigA2**, with the exception of a four amino acid variance in ASM151534.

#### 2.2.3. Aegerolysins from *Aspergillus terreus*

*Aspergillus terreus* Thom (1918) (*Eurotiales*) is a filamentous fungus found in soil worldwide. Although it was thought to be purely asexual, it is now known to be capable of sexual reproduction. This saprotrophic fungus is common in warmer climates such as the tropics and subtropics. It is found not only in soil, but also in habitats, such as decomposing vegetation and dust [21]. *A. terreus* is used in industry to produce important organic acids, such as itaconic acid, and enzymes. It was also the original source of the drug mevinolin (lovastatin), which is used to lower serum cholesterol. It can cause opportunistic infections in people with weakened immune systems [28].

Terrelysin (**Ter**) is an aegerolysin from *A. terreus*. The genome sequence was used to identify the **Ter** sequence based on homology with other known aegerolysins [28]. The recombinant protein **rTer** was purified and its mass of about 16 kDa was determined by MALDI-TOF MS [28]. Circular dichroism analysis shows that the secondary structure of the protein is predominantly β-sheet. Thermal denaturation results of **rTer** show that the protein retains the β-sheet confirmation up to 65 °C. Clinical identification of *A. terreus* in aspergillosis is limited to broad identification of lesions in affected organs by biopsy, radiological methods, or detection of fungal carbohydrates, which often does not allow species-specific identification. Due to the urgent need for diagnostic methods that allow early and specific identification of the pathogen, monoclonal antibodies to cytolytic hyphal exoantigens were generated [116]. Additionally, specific monoclonal antibodies against **rTer** were developed and used to quantify the native protein in hyphal and secretion fractions grown in liquid culture at various time points [28]. Time-course studies showed that **Ter** expression was highest during early hyphal growth and decreased dramatically after mycelial expansion [117]. Immunolocalization studies showed that **Ter** was not only localized in the cytoplasm of the hyphae, but was also present in greater amounts at the tip of the hyphae [117]. By ELISA, it was found that the highest concentrations of **Ter** were found in the culture supernatant during the early stages of hyphal growth, compared to later time points when hyphal growth and apical elongation were reduced. Because **Ter** does not possess a signal peptide, it was suggested that it might diffuse or be actively secreted during initial hyphal growth (i.e., apical elongation) by other, yet uncharacterized, processes [117]. These results were confirmed in cultures grown at both room temperature and 37 °C. These observations suggest that **Ter** may be a potential biomarker for *A. terreus* infections [117]. 

There is no MACPF-like partner protein gene encoded alongside the ***ter*** gene [92]. There are some differences in sequences around the putative intron splice site and at the C-terminus between **rTer** from ATCC 1012 and the genome sequences of NIH2624 (ASM14961) and IFO6365. 

#### 2.2.4. Aegerolysins from *Aspergillus oryzae*

*Aspergillus oryzae* (Ahlburg) E. Cohn (*Eurotiales*) is a fungus widely used in the traditional Japanese fermentation industry, including the production of soy sauce, sake, bean curd, and vinegar. Among filamentous fungi, *A. oryzae* is known to have outstanding potential for the production of various enzymes. In addition, developments in genetic engineering have led to the use of *A. oryzae* in modern biotechnology for the production of industrial enzymes [29]. 

Significant glucose repression was observed in solid-state cultures based on steamed rice and wheat. A promoter of a hemolysin-like gene (***hlyA***) from *A. oryzae* encoding an aegerolysin-like protein **AoHlyA** that is not repressed by glucose has been reported [118]. The ***hlyA*** promoter is activated by conditions or factors not restricted to sporulation. Four putative CCAAT sequences, six putative heat-shock transcription factor binding sequences (AGAAN), and two putative BrlA binding sequences (MRAGGGR) have been identified to affect transcription [118]. The CCAAT sequence is one of the most abundant *cis*-elements in the promoter regions of numerous eukaryotic genes. The heat shock transcription factor positively regulates the family of stress response genes, and the *brlA* gene is a regulator of conidiation in *A. oryzae*. Efficient expression of homologous and heterologous genes was shown under the control of the ***hlyA*** promoter stronger than under the commonly used *amyA* promoter [118,119,120].

Four genome sequences of *A. oryzae* are accessible in public databases: RIB40, 3.042 BCC7051, and 100-8. In total, three aegerolysins [92] and six putative MACPF-like proteins are encoded in the genome of *A. oryzae* strain RIB40. An identical protein sequence to **AoHlyA** is encoded in only one in another strain 3.042, which has two aegerolysins. Surprisingly, only one aegerolysin is encoded in strain 100-8, but it is present in all four strains with only one amino acid variation. Three additional sequences are found in BCC7051, one correspondingly short and two larger, one with a longer C-terminus and the other with a long N-teminus.

### 2.3. Fungal Aegerolysins from Ascomycota—Sordariomycetes

#### 2.3.1. Aegerolysins from *Beauveria bassiana*

The fungus *Beauveria bassiana* (Bals.-Criv.) Vuill., 1912 (*Hypocreales*) is considered both an entomopathogen and an endophyte. Entomopathogenic fungi can also perform other functions in nature, including endophytism, antagonism to plant diseases, promotion of plant growth, and root colonization [30,31]. The fungus *B. bassiana* is already known as a biopesticide for biological control of insect pests and as an asymptomatic companion of plants.

Structural modeling of beauveriolysin A (**BlyA**) suggests that it has a similar three-dimensional fold to other aegerolysins from fungi or bacteria [52]. **BlyA** showed the ability to bind specifically to lipid vesicles containing Chol in combination with both mammalian and invertebrate major membrane sphingolipids, SM, or CPE, respectively [52]. However, **BlyA** itself is neither hemolytic to bovine erythrocytes nor toxic to Sf9 insect cells [52]. Given its similarity to other aegerolysin pore-forming partnering proteins, **BlyB** was considered a candidate, but the possible formation of pores together with **BlyA** remains to be confirmed [52]. Similar protein pairs may be encoded by several entomopathogenic fungi from the class *Sordariomycetes* [52]. **BlyB** has been shown to best align the structures of some bacterial and mammalian MACPF proteins, rather than **PlyB [52]**. Two structures have been solved from *Proteobacteria*, for the Gram-negative insecticidal protein GNIP1Aa (PDB ID: 6FBM) from *Chromobacterium piscinae*, which has been shown to be specifically toxic to western corn rootworm larvae during feeding [121], and for Plu-MACPF (PDB ID: 2QP2) from a lethal pathogen of insects *Photorhabdus luminescens* [122]; no data are available on their partner proteins. The macrophage-expressed gene 1 protein (MPEG-1) or perforin-2 (PDB ID: 6U23 and analogs), which plays a role in the human immune system, was recently shown to function alone [123,124,125]. The murine complement component C9 (PDB ID: 6CXO) and the human complement component C8α chain are also involved in the immune system (PDB ID: 2RD7); in contrast to MPEG-1, proteins C8α and C9 are involved in the formation of a heterooligomeric protein-membrane attack complex (MAC) [123,124,126,127]. In conclusion, several proteins that served as templates for construction of a protein model of **BlyA**, several possible binary toxin partner proteins, and also organisms from which these proteins are derived, also have insecticidal (or immunological) properties [52]. **BlyA** could belong to a set of insecticidal proteins and metabolites that enable entomopathogenic fungi to have their specific lifestyle [52].

The sequences of **BlyA** are identical in all strains ARSEF 2860, D15, GCA_001682635.1, and JEF-007.

#### 2.3.2. Aegerolysins from *Trichoderma atroviride*

Although some *Trichoderma* species are known to be closely associated with higher plants (endophytes and plant growth promoters), *Basidiomycetes* (mushroom green mold disease), invertebrates (nematodes, marine sponges), and mammals (opportunistic pathogens of humans), most taxa have been recovered from dead wood and fruiting bodies of other fungi, suggesting that these are the original ecological niches of the fungus [32]. *Hypocrea atroviridis* Dodd et al. (*Trichoderma atroviride*) (*Hypocreales*) is a filamentous, cosmopolitan fungus commonly found in soil and has been isolated in both tropical and temperate climates. This mycoparasitic fungus is best known for its biocontrol capabilities in agriculture, interacting with phytopathogenic fungi and oomycetes, such as *Rhizoctonia solani* and *Botrytis cinerea*, which are pests of hundreds of crops [33,34,35]. 

The recently functionally characterized **Agl1** aegerolysin in this fungus shows a role in conidiation and antagonism. Gene expression analysis showed higher expression of **agl1** during conidiation and during growth in medium enriched with cell wall material from the plant pathogenic fungus *R. solani* as the sole carbon source. Expression of **agl1** was suppressed under iron-limiting conditions, whereas the **agl1** transcript was not detected during the interaction of *T. atroviride* with the prey fungi *B. cinerea* or *R. solani*. Phenotypic analysis of **agl1** deletion strain showed reduced conidiation compared with *T. atroviride* wild type, suggesting involvement of **Agl1** in conidiation. In addition, the **agl1** deletion strain showed reduced antagonism to *B. cinerea* and *R. solani* based on a secretion assay, although no difference was observed in direct interactions [128].

There is no bicomponent partner protein gene encoded alongside the **agl1** gene; there is only one amino acid variation between strains of *T. atroviride* IMI 206040, B10, F7, and P1 (ATCC 74058).

### 2.4. Fungal Aegerolysins from Ascomycota—Dothideomycetes

#### Aegerolysins from *Alternaria gaisen*

The cosmopolitan genus *Alternaria* (*Pleosporales*) contains many species of economic importance, including saprophytes, phytopathogens, and zoopathogens known to produce a variety of mycotoxins and allergens [36]. *Alternaria gaisen* Nagano ex Hara, 1920 (black spot of Japanese pear), occurs on the Japanese pear, *Pyrus pyrifolia*, especially the cultivar Nijisseiki, but also other cultivars of both Japanese and Chinese pears [37].

The accuracy and effectiveness of crop protection measures depends on correctly identifying of the pest of concern from the outset. However, the relatively invariant internal transcribed spacer sequence of structural ribosomal RNA that have been described and selected for fungal barcoding, for *Alternaria* species and strains did not allow discrimination of isolates of different *A. alternata*, *A. arborescens*, *A. brassicicola*, *A. gaisen*, *A. mali*, *A. tenuissima*, and *A. yaliinficiens*. When the partial genomic sequences of the putative aegerolysin **L152** were analyzed, the lineages were resolved, and they reflected the diversity previously suggested by the morphological evaluation of the sporulation patterns [36]. The presence of putative aegerolysin **L152** homologs in both phytopathogenic and non-phytopathogenic species argues against their involvement in pathogenesis. In addition, the putative aegerolysin gene is not located on any of the conditionally disposable chromosomes, so it is likely to be present in all small-spored *Alternaria* spp. and not only in the toxin producers [36]. Expression of **L152** in *A. gaisen* was strongly upregulated after light exposure, a stimulus that also triggers conidiophores to develop [36].

The only aegerolysin of *A. geisen* strain BMP2338, **L152**, also has a neighboring partner protein gene: **L152B**.

### 2.5. Bacterial Aegerolysins from Firmicutes

#### 2.5.1. Aegerolysins from *Bacillus thuringiensis*

*Bacillus thuringiensis* Berliner 1915 is a rod-shaped, Gram-positive, spore-forming, aerobic bacterium usually found in soils, grain dusts, dead insects, and water around the world. It has adapted its lifestyle as an efficient pathogen, synthesizing a large number of protein toxins that are effective against a variety of organisms in nature, including not only a broad range of insect orders but also nematodes, human pathogenic protozoans, animal, and human parasites. A molluscicidal activity against the snail pest has also been demonstrated [38,39]. 

*Bacillus thuringiensis* owes its insecticidal potential to several factors, and the main factor is the parasporal crystal containing a series of entomotoxins. The main *B. thuringiensis* entomotoxins, Cry (crystals), and/or Cyt (cytolytic) are pore-forming proteins, which are dissolved in the gastrointestinal tract of an insect after ingestion. A variety of different insecticidal proteins produced by *Bacillus* species is very rich. Cry toxins exert their membrane-permeabilizing activity on insect epithelial midgut cells after binding to a variety of protein receptors [129]. Among more than 300 members of the Cry toxins, a 13.6 kDa **Cry34Ab1** from *B. thuringiensis* (Bt strain PS149B1), is the only with the aegerolysin fold. It is composed of two β-sheets in a β-sandwich structure [130]. Similarly to aegerolysin/MACPF protein pairs described in oyster mushrooms, **Cry34Ab1** exerts its membrane activity and insecticidal action towards western corn rootworm in the presence of a 43.8 kDa partnering protein, **Cry35Ab1**. The latter protein has a toxin 10 (PF05431) C-terminal domain. **Cry34Ab1**/**Cry35Ab1** binary toxins have been transformed into maize plants under the commercialized events. After ingestion, they bind to the brush border membranes in the epithelial lining of western corn rootworm midgut, where they form pores. This leads to necrosis and ends with the insect’s death. The **Cry35Ab1** protein does not convey activity in the absence of **Cry34Ab1**, indicating that the smaller **Cry34Ab1** protein is critical for membrane binding and recruitment of **Cry35Ab1** to induce insecticidal effect [39,131,132,133,134,135]. Although until now the exact nature of **Cry34Ab1** protein receptor(s) in insect larva midgut membranes has not been determined [132,135], it appears that these receptors are unique and unrelated to other Cry toxins [136]. Further, although the **Cry34Ab1**/**Cry35Ab1** mixture was demonstrated to be slightly lytic to artificial lipid membranes in absence of proteins [133]; the lipid specificity seems to be low and the SM/Chol or CPE/Chol complexes cannot be regarded as receptor molecules, as in the case of oyster mushroom-derived aegerolysin/MACPF cytolytic insecticidal complexes [79]. Cristal structure of **Cry34Ab1**/**Cry35Ab1** binary toxin has been solved (PDB ID: 4JOX and 4JP0, respectively) [130]. 

Another characteristic feature of *B. thuringiensis* strains is the size of their extrachromosomal pool, with strains containing more than ten different plasmid molecules [137]. The size of these extrachromosomal molecules varies from two to more than 500 kb, and the number of encoded molecules can be as high as 17. For example, the complete sequence and organization of the transmitted 128 kb megaplasmid pBtoxis is known and has been compared to the large plasmid pXO1 of *B. antracis*; however, pBtoxis does not encode aegerolysin [138]. The main focus placed on these elements relates to the entomopathogenesis of their host strains and the plasmid-based genetic determinants of entomotoxins [139]. 

#### 2.5.2. Aegerolysins from *Clostridium bifermentans*

*Paraclostridium bifermentans* (Weinberg and Seguin 1918) Sasi Jyothsna et al., 2016 (*Clostridium bifermentans*) is an anaerobic, motile, Gram-positive bacterium capable of forming endospores. A particular subspecies, *C. bifermentans subsp. malaysia*, has been reported to have high mosquitocidal activity and no toxicity against mammals and various other nontarget organisms [40]. 

A number of proteins have been identified as potentially mosquitocidal. In 1998, two putative hemolysin-like proteins, **Cbm17.1** and **Cbm17.2**, were isolated from the bacterium *C. bifermentans* and later designed as aegerolysins [140]. Efficient expression of the ***cbm17.1*** and ***cbm17.2*** genes *C. bifermentans* was achieved by introducing them as His_6_-tagged proteins into *E. coli* and *B. thuringiensis* [140]. However, when biological activity of the recombinant proteins **Cbm17.1** and **Cbm17.2** was assayed, no toxicity to mosquito larvae and no hemolytic effect was observed [141,142]. The Cry operon of *C. bifermentans subsp. malaysia* was analyzed in 2014 and was found to contain four genes, two of which encode aegerolysins, **Cbm17.1** and **Cbm17.2**, and two of which encode larger protein partners, **Cry16Aa** (**cbm71**) and **Cry17Aa** (**cbm72**). All of these proteins (**Cry16Aa**/**Cry17Aa**/**Cbm17.1**/**Cbm17.2**) have been shown to be required for toxicity to larvae of mosquitoes of the genus *Aedes* [40]. 

Interestingly, in both *B. thuringiensis* and *C. bifermentans subsp. malaysia*, these aegerolysins and their partner proteins are encoded on an aegerolysin operon and are thus under the control of a single promoter [40].

### 2.6. Bacterial Aegerolysins from Proteobacteria

#### 2.6.1. Aegerolysins from *Alcaligenes faecalis*

Strains of the rod-shaped *Alcaligenes* species are found in soil, water, and in the environment associated with humans. The Gram-negative species *Alcaligenes faecalis* Castellani A and Chalmers AJ (1919) was first discovered in feces and named. Opportunistic infections in humans, usually urinary tract infections, are generally considered non-pathogenic. Nematicidal activity against *C. elegans* and *Meloidogyne incognita* has been reported [41]. 

The 16 kDa unit of the two-component insecticidal protein (**AfIP-1A**) is an aegerolysin which was identified in *A. faecalis* [143]. The structure of this aegerolysin was solved (PDB ID: 5V3S) [143]. **AfIP-1A/AfIP-1B** represents as a two-component insecticidal protein complex that exhibits high activity against the western corn rootworm. Transgenic maize plants expressing **AfIP-1A/AfIP-1B** show strong protection against rootworm damage [143]. Similarly to **Cry34Ab1**/**Cry35Ab1** binary toxins from *B. thuringiensis*, **AfIP-1A** binds directly to insects’ intestinal brush border membranes, whereas **AfIP-1B** does not. Binding of **AfIP-1B** occurs only in the presence of **AfIP-1A**, and is accompanied by the presence of stable, high molecular weight oligomers of **AfIP-1B** observed on denaturing protein gels. **AfIP-1A/AfIP-1B** complexes form pores in artificial lipid membranes [144], and likely share the same binding sites as **Cry34Ab1**/**Cry35Ab1** in brush border membranes of the western corn rootworm midgut epithelium [144]. The coordination of **AfIP-1B** binding by **AfIP-1A**, the similar structures of PDB ID: 5V3S (**AfIP-1A**) and PDB ID: 4JOX (**Cry34Ab1)**, and their shared binding sites in western corn rootworm intestinal tissues and cross-resistance suggest a similar role of **AfIP1A** and **Cry34Ab1** in receptor recognition and docking site for their cognate partners **AfIP-1B** and **Cry35Ab1**, respectively [144]. 

In contrast to *B. thuringiensis*, where **Cry34Ab1** and **Cry35Ab1** are plasmid-encoded, the two genes for **AfIP-1A** and **AfIP-1B** are chromosomally encoded as neighboring genes. Unlike other bicomponent toxin gene pairs identified in fungi, these two genes are oriented in the same direction in *A. faecalis* strains GCA_003521065 and GCA_005311025, whereas they are absent in three other strains: GCA_003813085, AN70, and YBY.

#### 2.6.2. Aegerolysins from *Pseudomonas aeruginosa*

*Pseudomonas aeruginosa* (Schröter 1872) Migula 1900 is a known ubiquitous opportunistic bacterial human pathogen that is widely distributed in patients with cystic fibrosis or acquired immune deficiency syndrome. It infects a remarkably evolutionarily diverse hosts, namely vertebrates, plants, and also insects; it is an important drug-resistant pathogen and has shown innate potential to promote plant growth and biological control in vitro [42,46]. 

The quorum-sensing receptor RhlR has been shown to trigger expression of the *P. aeruginosa* aegerolysin **RahU** as a complex with its cognate autoinducer N-butanoyl-homoserine lactone [145]. Expression of **rahU** gene is increased eightfold in the *P. aeruginosa* isolates with a nonmucoid phenotype, associated with the initial phase of lung infection. The biological role of **RahU** remains enigmatic, but it has been suggested that it may play a role in host–pathogen interaction [146]. **RahU** has been shown to associate with both the inner and outer membranes of the mucoid isolate [147]. **RahU** specifically interacts with CPE [148]; this CPE-specific interaction was demonstrated both on the artificial membranes containing physiologically relevant concentrations of CPE and on CPE-producing insect cell line. However, no interaction of **RahU** with the mammalian cell membrane was observed. The crystal structure of **RahU** (PDB ID: 6ZC1) showed the conserved aegerolysin folding as well as a ligand binding cavity [148]. Further mutational analysis revealed that residues within and around the binding site are important for the interaction of **RahU** with CPE-containing membranes, providing insights into the molecular discrimination of host cell membranes by **RahU** [148]. 

For **RahU**, there are no data on whether it acts alone or with a partner protein. There does not appear to be a gene for a MACPF-like partner adjacent on the chromosome of *P. aeruginosa* PAO1 or any other aegerolysin gene. In several strains, the **RahU** sequence is invariant; in more distant strains (according to whole genome comparison), such as PA7, only 77% identity (27 amino acid changes) was detected in the C-terminal shorter protein.

### 2.7. Aegerolysins from Insecta, Lepidoptera

#### Aegerolysins from *Pseudoplusia includes*

Loopers such as *Chrysodeixis includens* (Walker, 1858) (*Pseudoplusia includes*), *Trichoplusia ni* (Hübner, 1803), and *Helicoverpa zea* (Boddie, 1850) (Plusiinae) are important defoliators of soybeans, sunflowers, and crucifers, respectively, in countries of the Americas [47]. The polyphagous species larvae, such as the cabbage or soybean moth *C. includes*, feed on a wide range of plants [48]. 

Moths and butterflies (*Lepidoptera*) produce four types of hemocytes at the larval stage, which are usually identified by a combination of morphological, antigenic, and functional characteristics. Functionally, granulocytes phagocytize a variety of bacteria and other small organisms, and are required for the encapsulation of certain types of large foreign targets [48]. In last (fifth) instar larvae of *C. includens*, granulocytes comprise 60–70% of circulating hemocytes, are highly adhesive in vitro, and spread symmetrically on flat surfaces such as tissue culture plates. Circulating plasmatocytes differ in their ability to spread on foreign surfaces, and aegerolysin **P23**, named after the molecular mass band, serves as a marker for the subpopulation that is able to encapsulate foreign targets and respond to the plasmatocyte spreading peptide of the ENF peptide family (a consensus sequence of ENF at its amino termini), whose members act as potent activators of plasmatocyte adhesion-dependent activities in several *Lepidoptera* species [48]. **P23** showed no antibacterial or cytolytic activity against bacteria and mammalian erythrocytes [48]. However, it is noteworthy that the protein sequence of **P23** is most similar to the aegerolysin-like protein **TnAV2c gp029** encoded by *Trichoplusia ni* ascovirus 2c [49,50]. 

From the genome assembly of chromosome 6 of *P. includes*, it can be concluded that there are four aegerolysin genes in a row, 92, 52, 38, and 35% identical to P23, with one more gene between the first two. 

### 2.8. Aegerolysins from Virus, Ascoviridae

#### Aegerolysins from *Trichoplusia ni* ascovirus 2c (TnAV2c)

*Trichoplusia ni* ascovirus 2c (TnAV2c) (renamed to TnAV6a1) is an obligate viral pathogen of *T. ni*, *C. includes*, and *H. zea* larvae and selected other lepidopterans of the family *Noctuidae* [47,49,50]. Ascoviruses are a family of insect viruses with circular, double-stranded DNA genomes that cause chronic, lethal infections in insects of the order *Lepidoptera*. The name of this family of viruses is derived from the unique cellular pathology they cause, i.e., the formation of large, virion-containing vesicles 5–10 μm in diameter in the hemolymph of infected insects. While ascoviruses are poorly infectious by the oral route, they are spread among larvae in lepidopteran populations by parasitic wasps during oviposition. Certain parasitoid wasps pick up the virus after oviposition in an infected host and transmit the virions to new hosts during subsequent ovipositions [51].

High rates of infection with ascovirus TnAV-2 in a population of the corn earworm *H. zea* (as high as 74%) were found in a cotton growing area in Blackville, SC; observations showed that ascovirus infection alters the feeding behavior of *H. zea* larvae [49]. The complete genome sequence of TnAV-6a1 (TnAV-2c) was determined; the circular genome contains 165 open reading frames of greater than 180 bp [50]; ORF29 was identified as aegerolysin **TnAV2c gp029** [50,149]. Similar ORFs were also sequenced in another *T. ni* ascovirus **6b** [150], and another moth tobacco budworm *Chloridea virescens* (*Heliothis virescens*) ascovirus **3f** and **3h** [49,151]. RNA-sequencing was performed to compare the expression of TnAV-6a1 core genes during the first week of infection, with emphasis on the first 48 h, comparing transcript levels in major somatic tissues (epidermis, tracheal matrix, and fat body), the sites infected initially, with those of the hemolymph, where viral vesicles circulate and most replication occurs. In addition to the ascovirus core genes, several genes were identified in the contigs of the TnAV6a1 are unique. Only three of them were identified as highly expressed in hemolymph 48 h or 7 days after infection, namely the aegerolysin gene **ORF29** and two coding hypothetical protein genes. In somatic tissues, only the aegerolysin gene was identified as highly expressed 48 h after infection [152].

There is no MACPF-like protein encoded in this virus.

## 3. Sequences, Structures, and Structural Models of Aegerolysins

**Figure 2:**
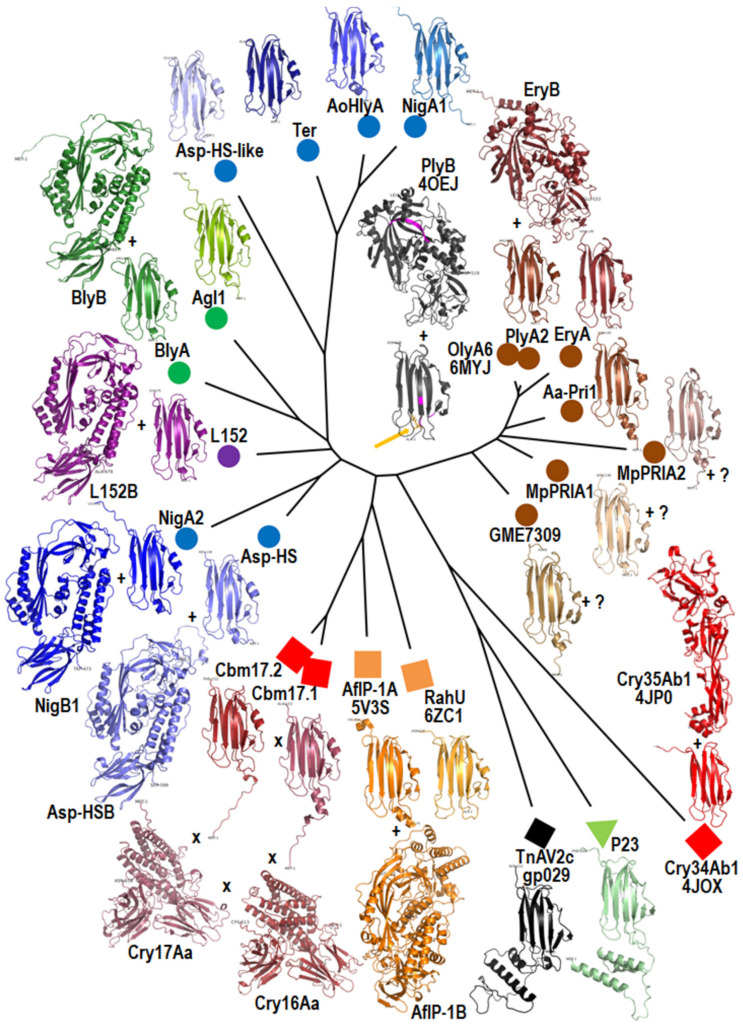
Phylogenetic tree of aegerolysins with some published information combined with their structures and models. Circles, proteins from fungi; A color legend is supplied for individual taxa: proteins from *Agaricomycetes* (OlyA6 and PlyB in grey and others in brown), *Eurotiomycetes* (in blue), *Sordariomycetes* (in green), and *Dothideomycetes* (in violet). Squares, bacteria; *Firmicutes* (in red), and *Proteobacteria* (in orange). Triangle, insects (in light green); diamond, virus (in black). Aegerolysin phylogeny (calculated by Mega [154]) is not following taxon phylogeny. Structures and AlphaFold2 calculated models of aegerolysins and protein partners [14]; presentation by PyMOL [62]. Aegerolysins: OlyA6/PlyA, ostreolysin A6/pleurotolysin A from *Pleurotus ostreatus*, PDB ID: 6MYJ; EryA, erylysin A, and PlyA2, pleurotolysin A2 from *Pleurotus eryngii*; Aa-Pri1, aegerolysin Aa-Pri1 from *Cyclocybe aegerita* (*Agrocybe aegerita*); MpPRIA1 and MpPRIA2, putative aegerolysin genes from *Moniliophthora perniciosa*; GME7309, *Lignosus rhinocerotis* aegerolsin-domain-containing protein; Asp-HS, Asp-hemolysin, and Asp-HS-like, Asp hemolysin-like from *Neosartorya fumigata* (*Aspergillus fumigatus*); Ter, terrelysin from *A. terreus*; AoHlyA, *A. oryzae* hemolysin; NigA1 and NigA2, nigerolysin A1 and A2 from *A. niger*; BlyA, beauveriolysin A from *Beauveria bassiana*; Agl1, *Hypocrea atroviridis* (*Trichoderma atroviride*) aegerolysin; L152, *Alternaria geisen* aegerolysin; Cry34Ab1 (Gpp34Ab1) 13.6 kDa insecticidal crystal protein PDB ID: 4JOX from *Bacillus thuringiensis*; Cbm17.1 and Cbm17.2, hemolysin-like protein Cbm17.1 and Cbm17.2 from *Paraclostridium bifermentans* (*Clostridium bifermentans*); AfIP-1A, two-component insecticidal protein 16 kDa unit, PDB ID: 5V3S, from *Alcaligenes faecalis*; RahU, RahU protein, PDB ID: 6ZC1, from *Pseudomonas aeruginosa*; P23, protein 23 from *Chrysodeixis includes* (*Pseudoplusia includes*); and TnAV6a1 gp029; ORF029 from *Trichoplusia ni* ascovirus 6a1 (2c). Partner proteins: PlyB, pleurotolysin B, PDB ID: 4OEJ; EryB, erylysin B; NigB1, nigerolysin B1, BlyB, beauveriolysin B; Cry35Ab1 (Tpp35Ab1), 43.8 kDa insecticidal crystal protein, PDB ID: 4JP0; Cry16Aa and Cry17Aa, pesticidal crystal-like protein Cry16Aa and Cry17Aa; and AfIP-1B, two-component insecticidal protein 77 kDa unit. The position of the only three conserved glycines is marked in the structure of OlyA6 (in magenta) (Figure S1), the position of the fragment of chemical structure of SM (18:1) (in orange and marked with arrow). SM, sphingomyelin; Typical signature Y/F-G-X2-F/Y-X6-G-G for fungal MACPF domain is marked in PlyB (magenta); x, Cry16Aa/Cry17Aa/Cbm17.1/Cbm17.2 act together; ?, no info about component B; +, having component B.

We have collected published experimental data on several aegerolysins and their analogs. The sequences of these aegerolysins were taken from published papers or, if not available there, from publicly available genomes; to avoid confusion, they were collected in File S1. The sequences of the aegerolysins ranged from 11 to 92% identical. Only three conserved amino acids, all three glycines, were observed in the alignment between them (Figure S1, star in magenta), and their position in the structure of OlyA6 (PDB ID: 6MYJ) was marked (Figure 2, in magenta). Two of them were located in two different loops on the same side of the molecule; the second loop contains an additional almost conserved glycine (except in **Cry34Ab1**), followed by a β-sheet containing the third conserved glycine. Due to the smallest side chain, the glycine can increase the local flexibility in the protein structure and the intrinsically destabilize β-sheet [153], possibly helping to accommodate the binding site. In the structure of **OlyA6**, the position of the fragment of the chemical structure of SM (18:1) is marked (Figure 2, in orange and orange arrow) [73].

Genome sequences from multiple strains are available for some of the species. Although we identified only one gene locus encoding the same aegerolysin sequence in the genome of the two laboratory monokaryotic model strains of *P. ostreatus* PC9 and PC15, the coding sequences of the aegerolysin protein may vary between other strains [52,53,54,55]. For example, the **OlyA6** protein differs in one amino acid (99% identical) from the genomic sequence **PlyA(PriA)**, and **PlyA** in six (95% identical) [52]. Perhaps it is also plausible to expect *P. eryngii*, a grassland litter decomposer consisting of at least five varieties such as *var. eryngii*, *ferulae*, *elaeoselini*, *thapsiae*, and *tingitanus*, which differ significantly with habitat distribution, host, and morphology, to contain analogs of the aegerolysins **PlaA2** or **EryA**. Similar variations in aegerolysin sequences have already been observed in some *Altenaria* strains [36]. Of the two aegerolysins described in *A. fumigatus*, an invariant **asp-HS-like** sequence was also present in six other strains; variation in one amino acid was observed in **asp-HS** between the strains, but **asp-HS** was also absent in one of the strains (sequence of component B from the same locus, **asp-HSB**, was only partial in the same strain). Variations in the aegerolysin sequences for **NigA1** were not observed in any of the nine other *A. niger* strains examined. A similar observation was made for **NigA2**, except for a variation of four amino acids in one of the strains. There are some differences in the sequences around the putative intron splice site and at the C terminus among the three *A. terreus* strains. Four genome sequences of *A. oryzae* are accessible in public databases; surprisingly, only one aegerolysin with only one amino acid variation is encoded in all four strains. Another strain has an additional gene identical to the strain with three aegerolysin genes. In the last strain, three additional sequences are found, one correspondingly short and two larger, one with a longer C-terminus and the other with a long N-terminus. The sequences of **BlyA** are identical in all four strains of *B. bassiana*. In the sequence of **Agl1**, there is only one amino acid variation among the four *T. atroviride* strains. One of the phylogenetically more distant strains, *P. aeruginosa* PA7 (according to whole genome comparison) revealed that **RahU** is only 77% identical to the PAO strain. Sequencing and annotation errors cannot be excluded.

For some species, all aegerolyins have been reasonably described, while others may have additional genes. One aegerolysin is encoded by the genome of *P. ostreatus*, *A. terreus*, *B. bassiana*, *T. atroviride*, *A. geisen*, *A. faecalis*, *T. ni* ascovirus 2c; two aegerolyins are encoded by *P. eryngii*, *A. niger*, *A. fumigatus* (except for one strain that has one aegerolysin), *P. aeruginosa*, and *C. bifermentans*; *A. oryzae* encodes one to four and *P. includes* four; *A. aegerita* and *M. perniciosa* encode six. No data for additional aegerolysin were found for *L. rhinocerotis*.

Five structures of aegerolysins have been solved: *P. ostreatus* **PlyA** (PDB ID: 4OEBA) and **OlyA6** (PDB ID: 6MYJ and some analogs), *B. thuringiensis* **Cry34Ab1** (Gpp34Ab1) (PDB ID: 4JOX), *A. faecalis* **AfIP-1A** (PDB ID: 5V3S), and *P. aeruginosa* **RahU** (PDB ID: 6ZC1). In addition, two structures of the two-component partner proteins *P. ostreatus* **PlyB** (PDB ID: 4OEJ) and *B. thuringiensis* **Cry35Ab1** (Tpp35Ab1) (PDB ID: 4JP0) have been solved, and furthermore, the membrane embedded pleurotolysin **PlyA**/**Plyb** pore with 13-fold symmetry (PDB ID: 4V2T) from *P. ostreatus* (Figure 1). Structures for other proteins were modeled (Figure 2) using the recently developed and highly accurate protein structure prediction program AlphaFold2 [14], providing a new dimension in the overview of this protein family.

Despite the low identity of the protein sequences (Figure S1), it is noteworthy that all aegerolysin models exhibit a tight β-sandwich fold similar to the actinoporin family of pore-forming proteins as identified by crystal structures (Figure 2, Graphical abstract) [60]. The presence of a short N-terminal helix was confirmed in the **AfIP-1A** structure (PDB ID: 5V3S). However, for some aegerolysins, such as **Aa-Pri1** and **Cbm17.1**, the presence of a shorter N-terminal helix could also be uncertain. Since some aegerolysins were isolated from fungi or bacteria, some of them were produced recombinantly from a DNA sequence, and for the rest only expression profiles were identified, there is already some uncertainty the exact protein sequences, especially at the ends. In addition, the determination of the N-terminal structure is sometimes less efficient due to flexibility, resulting in less accurate fold predictions. These short helices were also not supported by protein secondary structure predictions (Figure S2). However, two large additional α-helices were modeled for viral and insect aegerolysins (Figure 2 and Figure S2). 

## 4. Aegerolysin Binary Partner Proteins

Binary and quaternary cytolytic complexes of bacterial origin in which aegerolysin-like proteins are combined with larger, non-aegerolysin-like protein partner(s), described to date, include: **Cry16Aa**/**Cry17Aa**/**Cbm17.1**/**Cbm17.2** from *C. bifermentas subsp. malaysia* [40]; **Cry34Ab1**/**Cry35Ab1** from *B. thuringiensis* [130,134]; and **AflP-1A**/**AflP-1B** from *A. faecalis* [143]. In fungus *P. ostreatus*, **PlyA** forms a pore embedded in the membrane together with **PlyB** (Figure 1) [60]. Similar cytolytic effects were observed when **PlyB** was combined with other *Pleurotus*-derived aegerolysins, e.g., **OlyA6**, **PlyA2** and **EryA** [78]. These heteromeric aegerolysin-based cytolytic complexes have been exploited as potent biopesticides for specific pests, with **Cry16Aa**/**Cry17Aa**/**Cbm17.1**/**Cbm17.2** acting against *Aedes* mosquitoes, and **Cry34Ab1**/**Cry35Ab1, AflP-1A/AflP-1B, OlyA6**/**PlyB**, **PlyA/PlyB, PlyA2**/**PlyB**, or **EryA**/**PlyB** acting against *Coleoptera* species, especially the western corn rootworm. 

Unexpectedly, partner proteins can be classified into five groups (Table 2, Figure 2): (1) **PlyB** and **EryB** have a similar MACPF fold; (2) the remaining models, including **BlyB**, showed a reasonably good superposition; **BlyB** has been shown to best align the structure of bacterial GNIP1Aa, another MACPF domain-containing protein [52,121]; (3) the **Cry35Ab1** structure and (4) the **AfIP-1B** model do not superimpose with the **PlyB** structure or with each other; for **AfIP-1B**, the MACPF domain was found to be insignificant [52]; (5) **Cry16Aa** and **Cry17Aa** only superimpose with each other. 

Proteins containing a membrane-attack complex/perforin (MACPF) domain are transmembrane pore-forming proteins important for both human immunity and pathogen virulence. Little is known about the function of MACPF-like domain proteins in filamentous fungi and their taxonomic distribution. The number of putative MACPF proteins in a single fungal species ranges from zero to ten or more [155]. The identification and annotation of putative MACPF-like proteins is generally more error-prone because these genes have a higher number of introns [155]. The sequences can be divided into two groups. The proteins in the first group, such as **PlyB** or **EryB**, were assigned to the MACPF domain PF01823, which was confirmed by the 13-amino acid signature Y/F-G-X_2_-F/Y-X_6_-G-G typical of this domain, and they are grouped with human perforin. The second group of sequences is not recognized by the Pfam tool but still contains the typical MACPF/CDC signature, such as **BlyB** [155]. Fungal MACPF proteins probably contribute to various specific processes. While some of them were found in secretomes (without a typical signal sequence being recognized), others are intracellular, and some of them might be involved in pathogenesis, although probably not all [155]. The large number of introns, the sporadic taxonomic distribution, and the different number of MACPF proteins per fungal species might indicate the involvement of horizontal gene transfer mechanisms in specific ecological niches [155]. Putative MACPF-like domains were identified in the genomes of fungal species with different lifestyles; some of them are pathogenic, such as plant pathogen *M. perniciosa*, the caterpillar fungus *C. militaris*, or the nematode trap fungus *Arthrobotrys oligospora*. Some of the species are also saprophytic, such as the white rot fungus *P. ostreatus*, grassland-litter decomposers *P. eryngii*, saprophytic and food-producing *A. oryzae*, or saprophytic soil fungus *A. nidulans* [155]. 

However, the binary protein partner **Cry35Ab1** does not have a MACPF domain associated with it. Instead, it contains the C-terminal domain toxin 10 (PF05431), which is typical of a family of insecticidal crystal toxins of *Bacillus*, named after the insecticidal crystal toxin P42. **Cry35Ab1** also has an additional N-terminal ricin-type β-trefoil lectin domain (PF00652) (classified as insignificant only) [52,130]. 

**Cry16Aa** and **Cry17Aa** are delta endotoxins composed of three distinct structural domains, endotoxin N, M, and C, respectively. An N-terminal helical bundle domain is involved in membrane insertion and pore formation (PF03945), a central β-sheet domain is involved in receptor binding (PF00555), and the C-terminal β-sandwich domain interacts with the N-terminal domain to form a channel (PF03944). During sporulation, the bacteria produce crystals of delta endotoxins. When an insect ingests these proteins, they are activated by proteolytic cleavage. For all such proteins, the N-terminus is cleaved, and for some members, a C-terminal extension is also cleaved. After activation, the endotoxin binds to the intestinal epithelium and, by the forming cation-selective channels, causes cell lysis, which leads to death [137,156]. 

**Figure 3:**
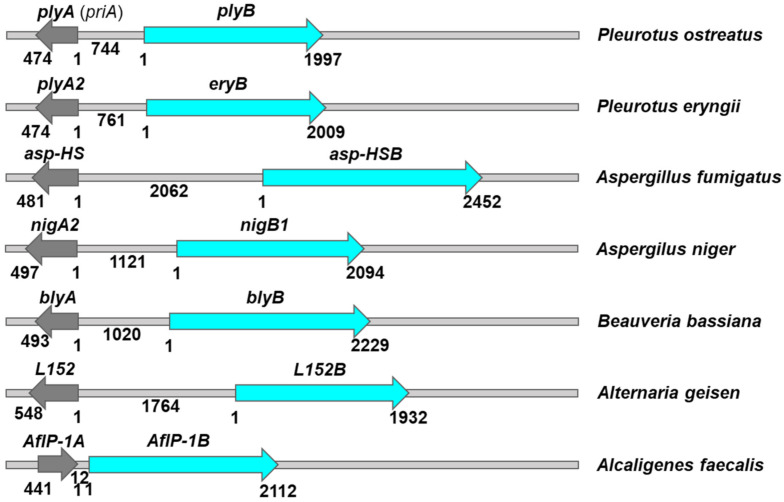
Aegerolysin gene loci in fungal and bacterial genomes. Grey arrows, aegerolysin genes; Cian arrows, membrane-attack-complex/perforin (MACPF)-like genes; plyA (priA) aegerolysin with plyB, pleurotolysin B gene from *Pleurotus ostreatus* PC9 whole genome sequence; [53]; PlyA2 with EryB from *P. eryngii* ATCC 907,970 [157]; Asp-HS with Asp-HSB from *Neosartorya fumigata* (*Aspergilus fumigatus*) Af293 [22]; nigA2 with nigB1, nigerolysin A2, and B1 gene *A. niger* CBS 513.88 [27]; blyA and blyB, beauveriolysin A and B gene from *Beauveria bassiana* ARSEF 2860 [158], L152 with L152B from *Alternaria geisen* BMP2338 [159], and AfIP-1A with AfIP-1B from *Alcaligenes faecalis* GCA_003521065; numbers, size of genes, and distance among genes in base pairs. Gene sizes and distances are scaled.

Surprisingly, it was observed in the genome of *P. ostreatus* that the two genes encoding the pair of pore-forming proteins also form a bidirectional pair with 5′–5′ orientation of **plyA** (**priA**) and **plyB** [91,92]. Similar gene pairs encoding putative bicomponent toxins have been observed previously, such as **Asp-HS** with **Asp-HSB**, **NigA2** with **NigB1**, and **BlyA** with **BlyB** from *A. fumigatus*, *A. niger*, and *B. bassiana*, respectively [52,92]. Moreover, such gene pairs have been also identified in other entomopathogenic fungi besides in *B. bassiana*, in the genomes of *Cordiceps militaris*, *Metarizium acridum*, *M. anisopliae*, *M. robertsii*, and *Ophiocordiceps sinensis*, but the involvement of these protein pairs in pore formation remains to be confirmed [52]. Here, we identified two additional putative pairs: **PlyA2** with **EryB** from *P. eryngii* and **L152** with **L152B** from *A. geisen* (Figure 3). The gene locations of the aegerolysin and putative MACPF-like genes in the genomes of some fungi and bacteria are schematically shown in Figure 3. A total of six fungal (putative) bicomponent toxins form a bidirectional gene pair with 5′–5′ orientation of the adjacent gene, and distances between the two genes vary (Figure 3). Clusters of genes encoding fungal secondary metabolites may also contain some reverse-oriented genes that may nevertheless show coordinate expression. In contrast to the fungal gene pairs, the **AfIP-1A**/**AfIP-1B** gene pair encoded on the bacterial chromosome has the same sense orientation (Figure 3). The bicomponent toxin gene pair **Cry34Ab1**/**Cry34Ab1** is known to be plasmid-encoded, but the variation of the plasmid in the bacterium *B. thuringiensis* is exceptionally high to see if these two genes are adjacent. The Cry operon in plasmid pCryO of *C. bifermentans subsp. malaysia* contains four genes downstream of the promoter pCyt: **Cry16Aa** is located 91 base pairs (bp) downstream, followed by **Cry17Aa**, which is located 426 bp downstream, followed by **Cbm17.1**, and 1022 bp downstream, followed by **Cbm17.2** [40]. Their placement at the same locus or under the control of the same promoter does not necessarily lead to their joint action, but rather indicates coordinated expression.

Some of the aegerolysins included in this review are also encoded in genomes that do not have an adjacent (MACPF domain-containing) partner protein; such aegerolysins are the fungal **EryA**, **Aa-Pri1**, **Asp-HS-like**, **Ter**, **AoHlyA**, and **NigA1**, bacterial **RahU**, insects **P23** and the ascoviral **TnAV2c gp029** (Table 2). For some of them, insufficient data are available, such as for the **MpPRIA1**, **MpPRIA2**, and **MpPRIB**, because the sequenced countings are too short, or no genomic data were found for **GME7309** (Table 2). However, a combined action with MACPF domain-containing proteins encoded elsewhere in the genome cannot be excluded, as some aegerolysins, such as **PlyA2** and **EryA** from *P. eryngii*, have been shown to exhibit cytolytic activity when combined with their non-native partner with component B, **PlyB**, from *P. osteratus* [78].

## 5. Lifestyle of Organisms Encoding for Aegerolysins and Putative Function of Aegerolysins

The aegerolysins listed in this review belong to several taxonomically diverse organisms; in fungi, they belong to four species of mushrooms and seven filamentous fungi, and they were also identified in four bacterial species, moth, and ascovirus (Table 1). Most of these species, which have very different lifestyles, combine several life forms or hosts; there are many transitions between these lifestyles or switches to other lifestyles. Each species belongs to a particular niche, some are very specific, and others are very ubiquitous and cosmopolitan. Some of these species could or would share the same niche, others would not. A wide range of lifestyles has been identified for organisms containing aegerolysins (Table 1): saprotrophic (white rot, grassland-litter decomposer, plant debris in compost piles, hay and straw stacks), entomopathogenic, mosquito larvicidal, endophytic, mycoparasitic (including oomycetes), nematocidal, plant pathogenic, facultative biotrophic, defoliating (feeding on a wide variety of plants), obligate pathogenic to moth larvae, ubiquitous opportunistic pathogenic to a wide range of hosts: humans, vertebrates, plants, insects, nematodes, mollusks, protozoans, or animal and human parasites. 

In the majority of the species listed, saprotrophic lifestyle predominates, as in the white rot fungi *P. ostreatus*, *L. rhinocerotis*, and *A. aegerita*, or in decomposers of grassland litter such as *P. eryngii* or *Aspergilli* living on plant debris in compost piles and stacks of highly moist hay and straw. Just because saprotrophic organisms feed primarily on dead or decaying matter, it is easy to confuse the saprotrophic niche with an environment where there is no competition. However, niche, such as wood decay, is one of the most competitive environments. Wood decay fungi have historically been characterized as either white rot fungi, which degrade all components of plant cell walls, including lignin, leaving a white color and rotting texture of the remaining crystalline cellulose, or brown rot fungi, which leave the lignin largely intact. However, genomic analyzes have shown that the prevailing paradigm of white rot and brown rot cannot to capture the diversity of wood decomposition mechanisms by fungi [15]. There is usually one fungal species that dominates a rotting trunk. The ability of fungi to colonize and decompose wood may be strongly influenced by wood-inhabiting bacteria that grow on readily usable compounds released by fungal enzymes. However, it is not known how white rot fungi (or other saprotrophic fungi) cope with the presence of potentially competing bacteria [160]. According to Gause’s principle of competitive exclusion, two species with identical niches (competing for a single resource) cannot coexist indefinitely [161]. In primary resource acquisition, fungi compete for influence over a resource but do not directly challenge others, whereas secondary resource acquisition involves combative interactions [162].

Although wood decomposition is largely driven by microbial activities, invertebrates, such as insect larvae and nematodes, also play important roles in both temperate and tropical environments. Key mechanisms include enzymatic digestion by endogenous enzymes and those produced by their symbionts, substrate modification by tunneling or fragmentation, biotic interactions, and nitrogen fertilization through nitrogen fixation by endosymbionts and free-living bacteria [163]. The effects of individual invertebrate species can be accelerating or inhibiting, but the cumulative effect of the entire community generally accelerates wood decomposition, at least in the early stages of the process [163]. Some taxa appear to be particularly influential in promoting wood decomposition, such as large wood-boring beetles (*Coleoptera*) and termites (*Termitoidae*), especially those cultivating fungal gardens [163].

In certain situations, saprophytic fungi can even become pathogenic; it seems they have tools at the ready just in case. For example, because *Aspergilli* are able to live in compost or haystacks at elevated temperatures, they can also become (opportunistic) pathogens for mammals. Mushrooms can be poisonous to organisms (such as rodents, humans, or snails) that choose to consume them in excessive quantities (*P. ostreatus* [164]), facultative pathogens on some other tree species *(A. aegerita*), or facultative biotrophs on roots of herbaceous plants (*P. eryngii*). 

Fungal, but also bacterial entomopathogens, are largely facultative parasites and play an important role in controlling the density of insect populations in nature; some of these organisms have been used for biological control of insect pests in agriculture. The entomopathogenicity of fungi has evolved several times; convergent evolution led to the formation of similar protein families in different species to adapt to insect hosts [165]. The host specificity of insect pathogens is related to the characteristics of the fungal genome and lifestyle. Species with a narrow host range usually have reduced protein family sizes, but retain the ability to reproduce sexually compared to generalists [165]. Similar to plant pathogens, entomopathogenic fungi encode a number of effector-like proteins in their genomes, suggesting the presence of analogous gene-for-gene relationships in fungus-insect interactions [165]. Insect pathogens employ different strategies to invade the host and evade its immune response [165]. The pattern of competition among entomopathogens for insect individuals remains unclear. For example, empirical competition for hosts or niches between the species of *B. bassiana* and *M. robertsii* was studied; insects were largely killed and mycotized by *M. robertsii*, regardless of the initial co-inoculation dose and infection order [166]. Thus, these results support the pattern of competitive exclusion between insect pathogenic fungi that occurs from outside to inside of insect hosts. However, parasexual recombination between the compatible strains apparently occurs after coinfection [166]. The sexual cycle of the heterothallic *Metarhizium* and *Beauveria* species rarely occurs in the field. Instead, parasexual recombination takes place between compatible strains through the process of hyphal fusion, heterokaryosis, and mitotic crossing-over for limited genetic recombination [167,168]. Thus, it seems that exclusive competition may occur between incompatible strains, but recombination arises from the compatible strains of fungal insect parasites [166]. Some entomopathogenic fungi, such as *B. bassiana* and *T. atroviride*, are also considered as endophytes, protecting plant roots by being mycoparasitic (including oomycetes) [169,170].

It has been shown that *P. ostreatus* paralyzes nematodes from the genera *Caenorhabditis*, *Diploscapter*, *Oscheius*, *Rhabditis*, *Pristionchus*, *Panagrellus*, *Acrobeloides*, *Cephalobus*, *Mesorhabditis*, and *Pelodera*, and consumes prey [15]. The unclear nematocidal mechanism of *P. ostreatus* triggers massive calcium influx and rapid cell necrosis in the neuromuscular system of *C. *elegans** via the sensory cilia of this nematode, a mechanism of action is common against all nematodes [15]. *Pleurotus eryngii* also elicits a nematocidal action. Several bacterial pathogens, including *P. luminescens* [171] and *B. thuringiensis* [172], are also known to induce necrotic responses in *C. elegans*. Nematopathogenic fungi have been identified as a basis for biological control of root-knot nematodes [173].

Membrane lipids or their specific combinations have been often associated with the binding of aegerolysins. The most common was the combination of CPE/Chol or SM/Chol. Aegerolysins sometimes interact with both of these lipid combinations, sometimes excluding one another, or bind to CPE alone, but never to free SM. They can also bind a combination of other lipids like CAEP/POPC/Chol or CL/DPPC/Chol (Table 2). The molar ratio of lipids may be important. For some of aegerolysins an unknown protein receptor in the lipid membrane has been proposed, or for others binding was not studied. Different lipids or their combination may define the binding target of aegerolysins because different organisms have different composition of major membrane lipids, particularly the sphingolipids. 

Sphingolipids are a numerous and versatile group of lipids in eukaryote and in some bacterial taxa. These amphipathic molecules can be abundant components of cell membranes, and their sphingosine-based degradation products are involved in many physiological processes, including signaling [77]. One of the major sphingolipids in mammalian cell membranes that has been extensively studied is SM, which plays a central role in the formation of lipid rafts and ordered membrane domains, as well as cell signaling processes and regulation of plasma membrane and cholesterol homeostasis [77]. SM is also the major sphingolipid of nematode *C. elegans*. CPE is the major sphingolipid of *Oomycetes*, such as *Pythium ultimum*, *Phytophthora infestans*, and *P. capsici*, as well as of some flagellated protozoa, and the major sphingolipid in the lipid membranes of various invertebrates, including insects. In some invertebrates, CPE is combined with SM, such as mosquitoes *Aedes aegypti*, *Culex quinquefasciatus*, or moths as *Manduca sexta*. In some *Bacteroidetes*, CPE is combined with ceramide phosphoglycerol, such as in *Flectobacillus major* and *Bacteroides fragilis*. CAEP is a CPE analog of aquatic invertebrates, such as mollusks and cnidarians [77]. 

The aegerolysins appear to have a (different?) function(s) associated with the specific stage of their development; it seems possible that they serve the interaction between organisms in the environment. Based on their sequence and binding diversity, it appears that these proteins might serve to scan the membranes of potential competitors or hosts in the niche. After binding to a specific lipid or lipid combination, or to yet unknown proteins receptors in lipid membranes, some of them may serve to dock various partner proteins from the organism of origin or perhaps from the target organism after the proteolytic cleavage. It appears that aegerolysins represent a part of adaptive response to the environment, by sensing lipid components of organisms in their environment and excluding competitors by forming two-component pores as a kind of primitive “immune interaction.” Some partner proteins have a similar (MACPF) fold to some molecules, such as MPEG-1, or proteins C8 and C9, which are involved in the formation of a heterooligomeric protein-membrane attack complex (MAC), and have a function in mammalian immune defense [126,127]. It is possible that organisms such as hemibiotrophic plant pathogen *Moniliophthora perniciosa* can increase their flexibility in lipid sensing with additional copies (six) of aegerolysin genes (if differently expressed). 

Horizontal gene transfer often plays a role in shaping the mechanisms by which a pathogen evades, counteracts, mimics, subverts, and manipulates the host [174]. Plasmid-encoded genes for aegerolyins and partner proteins of the ubiquitous, aerobic, and spore-forming organism, such as *B. thuringiensis*, which is an opportunistic pathogen for very diverse hosts, including vertebrates, plants, insects, nematodes, mollusks, protozoa, animal and human parasites, appear to be particularly susceptible to their dissemination. Especially in combination with pore formation, which may facilitate the presence or uptake of heterologous DNA molecules or their parts present in the environment. It is also known that *B. thuringiensis* harbors a variety of transposable elements, including insertion sequences and transposons [139,175]. In addition, it has been already shown that partial genes can serve as a source of novel Cry toxins [176]. Other possible vectors are (DNA) viruses. Whether *Trichoplusia ni* and *Heliothis virescens* ascoviruses acquired aegerolysin-like genes from their insect hosts or vice versa is unclear, but their presence in both, which may indicate horizontal gene transfer, suggests that this family of structurally similar proteins is more widespread and functionally diverse than previously thought [48].

## 6. Conclusions and Future Research

It seems possible that aegerolysins enable an adaptive response to the environment by sensing (lipid) components of the environment and excluding competitors by forming two-component pores. However, many questions remain. 

(1) Given the low identity of the protein sequences, the conserved structure of the aegerolysin molecules seems to be most important for their function. It remains to be clarified how the (lipid) binding interaction is specified and what contributes to the differences in specificity.

(2) Some organisms encode multiple aegerolysin copies. Some of the aegerolysins recruit partner proteins to function in pore formation. It is interesting to show whether other aegerolysin copies from the same organism also recruit the same component B protein. It has been shown in the laboratory that component B exchange is possible between closely related species. It is a challenge to show whether such acquisition of component B also occurs in nature, and how homologous the component B must be.

(3) If other copies of aegerolysin do not acquire component B, the question arises as to what their function is; perhaps they serve as decoys.

(4) Some of the partner proteins are MACPF domain-containing proteins. Some of the microbial or mammalian MACPF/CDC proteins form pores without binding to aegerolyins because they have their own C-terminal binding domain. It has been shown that some of them can be cleaved by unknown proteases [177]. It would be very interesting to show that the C-terminal binding domain of a MACPF domain-containing protein from the target organism can be replaced by aegerolysin from the competitor, which would then lead to a counteraction. 

## 7. Materials and Methods

### 7.1. Literature Search

Search for publications on aegerolyins was performed by Scopus title-abstract-keywords search (TITLE-ABS-KEY (aegerolysin*) [178]; 43 documents were initially identified: [1,2,3,4,5,6,7,8,9,10,11,28,36,48,52,63,64,65,66,67,72,75,77,78,79,80,81,82,84,87,91,92,93,101,104,105,114,128,130,144,145,147,148]; *Clostridium bifermentas* aergerolysins were added to the collection.

### 7.2. Comparative Genomics

Mining for the number aegerolysins from fungi was performed on the web portal MycoCosm, mining for the number aegerolysins from (bacteria and archaea) on the web portal Ensembl Bacteria, databases were searched for domain PF06355 named aegerolysin [12,13,179,180]. The genomes of were screened for differences in aegerolysin sequences and adjacent MACPF-like genes (PF01823) [12,13,179,180,181]. We found a genome of a single strain available for: *Agrocybe aegerita* AAE-3 [12,17], *Alternaria geisen* BMP2338 [12,159], *Trichoderma atroviride* [12,182], *Lignosus rhinocerotis* [12,106], *Moniliophthora perniciosa* FA553 [12,183], *Pleurotus eryngii* ATCC 90,797 [12,157], and *Pseudoplusia includens* chromosome 6 PRJEB38103 (GCA_941860345.1). Multiple strains were available for: *Alcaligenes faecalis*: GCA_003521065, GCA_005311025, GCA_003813085, AN70 (GCA_004319585), and YBY (GCA_003122065) [13]; *Aspergilus fumigatus*: Af293 [12,22,184]; A1163 [12,22,185,186,187]; GCA_002234955.1, GCA_002234985.1, Z5, and var. RP-2014 [181]; *Aspergillus niger*: CBS 513.88 [12,27,184], (*lacticoffeatus*) CBS 101883, (*phoenicis* Corda) Thom ATCC 13,157 [12,188], ATCC 1015 [12,189], NRRL3 [12,188,190], ATCC 13496, ATCC 64,974 N402, GCA_001515345.1, GCA_002211485.2, and GCA_000002855.2 [181]; *Aspergilus terreus*: NIH 2624 [12,184], and IFO6365 [181]; *Aspergilus oryzae*: RIB40 [12,191], 100-8, 3.042, and BCC7051 [181]; *Beauveria bassiana*: ARSEF 2860 [12,158]; D15, GCA_001682635.1, and JEF-007 [181]; *Pleurotus ostreatus* PC9 v1.0 and PC15 v2.0 [12,53,54,55], and *Pseudomonas aeruginosa* PAO1 and PA7 [13,192]. The aegerolysin protein sequences of different strains were aligned using the ClustalW web tool [193].

### 7.3. Phylogenetic Analysis

Phylogenetic analysis of ClustalW aligned aegerolysins and inferred by the Maximum Likelihood method was performed using Molecular Evolutionary Genetics Analysis, version 11 (MEGA11) [154,194].

### 7.4. Protein Structure Prediction

The deep learning algorithm AlphaFold2 was used for protein structure modeling [14]. UCSF ChimeraX version 1.3 (8 December 2021) and UCSF ChimeraX version 1.4 (3 June 2022) were used to run AlphaFold version 2 in conjunction with a free and accessible platform for protein folding ColabFold [195,196,197,198,199]. 

### 7.5. Protein Structure Presentation

Cartoon representation of the structures was done using PyMOL, version 2.2.0 [62]. Amino acid at N- and C-terminus of the model are marked.

### 7.6. Alignment of Proteins and Prediction of Secondary Structure

A multiple sequence alignment was performed with the workbench editor Jalview version 2.11.2.4 using the Clustal W version 2.1 /Clustal X algorithm [200,201]. The protein secondary structure prediction server JPred4, accessible via Jalview, was used to predict the secondary structure of proteins [202].

## Figures and Tables

**Table 1:** Organisms that contain aegerolysins according to taxonomy and lifestyles.

Organism Name	Other Names	Taxonomy	Lifestyle/Niche	Reference
		Fungi		
*Pleurotus ostreatus*	Oyster mushroom Hiratake	*Agaricomycotina* *Agaricales*	Saprotroph White rot Nematocidal	[15]
*Pleurotus eryngii*	King oyster or trumpet or brown mushroom Boletus of the steppes French horn mushroom Aliʻi oyster	*Agaricomycotina* *Agaricales*	Saprotroph Grassland-litter decomposer Facultatively biotrophic Nematocidal	[16]
*Cyclocybe aegerita*	*Agrocybe aegerita* Poplar mushroom Tea tree mushroom Cha shu gu Yanagi-matsutake Sword-belt mushroom Velvet pioppini	*Agaricomycotina* *Agaricales*	Saprotroph Weak white rot on hardwoods Facultatively pathogenic	[17]
*Moniliophthora perniciosa*	*Crinipellis perniciosa* Witches’ broom disease	*Agaricomycotina* *Agaricales*	Hemibiotrophic plant pathogen Broad range of host	[18]
*Lignosus rhinocerotis*	Tiger milk mushroom	*Agaricomycotina* *Polyporales*	Saprotroph White rot	[19]
*Neosartorya fumigata*	*Aspergillus fumigatus*	*Eurotimycetes* *Eurotiales*	Saprotroph Ubiquitous in soil and compost Human (opportunistic) pathogen	[20,21,22,23]
*Aspergillus niger*		*Eurotimycetes* *Eurotiales*	Saprotroph Ubiquitous in soil and compost Human opportunistic pathogen	[20,21,24,25,26,27]
*Aspergillus terreus*		*Eurotimycetes* *Eurotiales*	Saprotroph Human opportunistic pathogen	[20,21,28]
*Aspergillus oryzae*		*Eurotimycetes* *Eurotiales*	Saprotroph	[20,21,29]
*Beauveria bassiana*		*Sordariomycetes* *Hypocreales*	Entomopathogen Endophyte Soil and insects	[30,31]
*Hypocrea atroviridis*	*Trichoderma atroviride*	*Sordariomycetes* *Hypocreales*	Mycoparasitic (including oomycetes) Cosmopolitan, soil	[32,33,34,35]
*Alternaria geisen*	Black spot of Japanese pear	*Dothideomycetes* *Pleosporales*	Plant pathogen	[36,37]
		**Bacteria**		
*Bacillus thuringiensis*		*Firmicutes*	Ubiquitous opportunistic pathogen on vertebrates, plants, insects, nematodes, mollusks, protozoan, animal, and human parasites. Soils, grain dusts, dead insects, water Aerobic and spore-forming	[38,39]
*Paraclostridium bifermentans* *subsp. malaysia*	*Clostridium bifermentans* *subsp. malaysia*	*Firmicutes*	Anaerobic, forming endospores Mosquito larvicidal	[40]
*Alcaligenes faecalis*		*Proteobacteria*	Soil, water, environments associated with humans Human opportunistic pathogen Nematocidal	[41]
*Pseudomonas aeruginosa*		*Proteobacteria*	Ubiquitous opportunistic pathogen on: humans, vertebrates, plants, and insects	[42,43,44,45,46]
		**Insecta**		
*Chrysodeixis includens*	*Pseudoplusia includes*	*Lepidoptera* *Noctuidae*	Plant pest (defoliator) Larvae feed on a wide range of plants	[47,48]
		**Viria**		
*Trichoplusia ni* ascovirus 6a1	*Trichoplusia ni* ascovirus 2c	*Varidnaviria* *Ascoviridae*	Obligate pathogen *P. includens* moth larvae	[47,49,50,51]

**Table 2:** List of published aegerolysins.

Short Name	Aegerolysin/ Structure	Membrane Receptor	Function	Partner Protein Short Name	Partner Protein/ Structure	Organism	Reference
**PlyA**	Pleurotolysin A PDB ID: 4OEBA	SM/Chol	n.d.	**PlyB**	Pleurotolysin B PDB ID: 4OEJ Membrane embedded PlyA/PlyB pore PDB ID: 4V2T	*Pleurotus ostreatus*	[59,61]
**OlyA**	Ostreolysin A	SM/Chol CPE/Chol Lipid rafts	Involvement in mushroom fruiting Anticancer (+PlyB)	**PlyB**	Pleurotolysin B PDB ID: 4OEJ	*Pleurotus ostreatus*	[63,64,65,66,67,68,69,70,71,76,80,84,85,86,102,104]
**OlyA6**	Ostreolysin A6 PDB ID: 6MYJ	SM/Chol CPE/Chol CAEP/POPC/Chol Lipid rafts	Insecticidal (+PlyB)	**PlyB**	Pleurotolysin B PDB ID: 4OEJ	*Pleurotus ostreatus*	[72,73,74,75,78,79,80,81,82,83,87]
**rOly**	Recombinant ostreolysin	Lipid rafts?	Antiproliferative Pro-apoptotic	n.d.	n.d.	*Pleurotu ostreatus*	[88,89,90]
**PlyA2**	Pe.PlyA/Pleurotolysin A2	SM/Chol CPE/Chol CPE Lipid rafts	Insecticidal (+PlyB)	**EryB**	Erylysin B	*Pleurotus eryngii*	[15,79,80,93,94,95,96]
**EryA**	Erylysin A	CPE/Chol CL/DPPC/Chol	Insecticidal (+PlyB) Inhibition of cytokinesis	No	No	*Pleurotus eryngii*	[78,80,96,98,99]
**Aa-Pri1**	Aegerolysin Aa-Pri1	n.d.		No	No	*Agrocybe aegerita*	[63,100]
**MpPRIA1**	Putative aegerolysin	n.d.		**MpPLYB**?	n.d.	*Moniliophthora perniciosa*	[101]
**MpPRIA2**	Putative aegerolysin	n.d.		**MpPLYB**?	n.d.	*Moniliophthora perniciosa*	[101]
**GME7309**	GME7309_g aegerolysin-domain-containing protein	n.d.		n.d.	n.d.	*Lignosus rhinocerotis*	[105]
**Asp-HS**	Asp-hemolysin	Oxidized low-density lipoproteins	Cytotoxic effects on murine macrophages and vascular endothelial cells Induce cytokine genes	**Asp-HSB**	n.d.	*Aspergillus fumigatus*	[107,108,109,110,111,112,113,114,115]
**Asp-HS-like**	Asp hemolysin-like	n.d.	n.d.	No	No	*Aspergillus* *fumigatus*	[114]
**NigA1**	Nigerolysin A1	n.d.	n.d.	No	No	*Aspergillus niger*	[91,92]
**NigA2**	Nigerolysin A2	CPE/Chol	n.d.	**NigB1**	Nigerolysin B1	*Aspergillus niger*	[91,92]
**Ter**	Terrelysin	n.d.	n.d.	No	No	*Aspergillus terreus*	[28,116,117]
**AoHlyA**	*Aspergillus oryzae* hemolysin	n.d.	n.d.	No	No	*Aspergillus oryzae*	[118,119,120]
**BlyA**	Beauveriolysin A	SM/Chol CPE/Chol	n.d.	**BlyB**	Beauveriolysin B	*Beauveria bassiana*	[52]
**Agl1**	*Trichoderma atroviride* aegerolysin	Conidiation Antagonism	n.d.	No	No	*Trichoderma atroviride*	[128]
**L152**	*Alternaria geisen* aegerolysin	n.d.	n.d.	**L152B**	n.d.	*Alternaria geisen*	[36]
**Cry34Ab1** **(Gpp34Ab1)**	13.6 kDa Insecticidal crystal protein PDB ID: 4JOX	Unknown protein receptor	Insecticidal (+Cry34Ab1)	**Cry35Ab1** **(Tpp35Ab1)**	43.8 kDa insecticidal crystal protein PDB ID: 4JP0	*Bacillus thuringiensis*	[39,129,130,131,132,133,134,135,136]
**Cbm17.1**	Hemolysin-like protein Cbm17.1	n.d.	Insecticidal (+Cry16Aa/Cry17Aa/ Cbm17.2)	**Cry16Aa, Cry17Aa, Cbm17.2**	Pesticidal crystal-like protein Cry16Aa and Cry17Aa, Hemolysin-like protein Cbm17.2	*Clostridium bifermentans*	[40,140,142]
**Cbm17.2**	Hemolysin-like protein Cbm17.2	n.d.	Insecticidal (+Cry16Aa/Cry17Aa/ Cbm17.1)	**Cry16Aa, Cry17Aa, Cbm17.2**	Pesticidal crystal-like protein Cry16Aa and Cry17Aa, Hemolysin-like protein Cbm17.1	*Clostridium bifermentans*	[40,140,142]
**AfIP-1A**	Two-component insecticidal protein 16 kDa unit PDB ID: 5V3S	Unknown protein receptor AfIP-1A/AfIP-1B membrane pore	n.d.	**AfIP-1B**	Two-component insecticidal protein 77 kDa unit	*Alcaligenes faecalis*	[143,144]
**RahU**	RahU protein PDB ID: 6ZC1	CPE/Chol	n.d.	No	No	*Pseudomonas aeruginosa*	[145,148]
**P23**	n.d.	n.d.	n.d.	No	No	*Pseudoplusia includes*	[48]
**TnAV2c** **gp029**	n.d.	n.d.	n.d.	No	No	*Trichoplusia ni ascovirus 2c*	[49,50,149,150,152]

Chol, cholesterol; CPE, Ceramide phosphoethanolamine; SM, sphingomyelin; CAEP, ceramide aminoethylphosphonate; DPPC, dipalmitoylphosphatidylcholine; CL, cardiolipin; POPC, phosphatidylcholine; DPPC, dipalmitoylphosphatidylcholine; n.d., no data; ?, not enough data.

## Data Availability

No new data were created or analyzed in this study. Data sharing is not applicable to this article.

## Supplementary Materials

The following supporting information can be downloaded at: https://www.mdpi.com/article/10.3390/toxins14090629/s1, Supplementary File. Sequences of aegerolysins and partner proteins; Figure S1. Alignments of amino acid sequences of aegerolysins; Figure S2. Prediction of secondary structures of aegerolysins.

## References

[1] Berne S., Lah L., Sepčić K. (2009). Aegerolysins: Structure, function, and putative biological role. Protein Sci..

[2] Butala M., Novak M., Kraševec N., Skočaj M., Veranič P., Maček P., Sepčić K. (2017). Aegerolysins: Lipid-binding proteins with versatile functions. Semin. Cell Dev. Biol..

[3] Nayak A.P., Green B.J., Beezhold D.H. (2013). Fungal hemolysins. Med. Mycol..

[4] Novak M., Kraševec N., Skočaj M., Maček P., Anderluh G., Sepčić K. (2015). Fungal aegerolysin-like proteins: Distribution, activities, and applications. Appl. Microbiol. Biotechnol..

[5] Ota K., Butala M., Viero G., Dalla Serra M., Sepčić K., Maček P., Anderluh G., Gilbert R. (2014). Fungal MACPF-like proteins and aegerolysins: Bi-component pore-forming proteins?. Sub-Cellular Biochemistry.

[6] Yamaji-Hasegawa A., Hullin-Matsuda F., Greimel P., Kobayashi T. (2016). Pore-forming toxins: Properties, diversity, and uses as tools to image sphingomyelin and ceramide phosphoethanolamine. Biochim. Biophys. Acta Biomembr..

[7] Kishimoto T., Ishitsuka R., Kobayashi T. (2016). Detectors for evaluating the cellular landscape of sphingomyelin- and cholesterol-rich membrane domains. Biochim. Biophys. Acta Mol. Cell Biol. Lipids.

[8] Hullin-Matsuda F., Makino A., Murate M., Kobayashi T. (2016). Probing phosphoethanolamine-containing lipids in membranes with duramycin/cinnamycin and aegerolysin proteins. Biochimie.

[9] Hullin-Matsuda F., Murate M., Kobayashi T. (2018). Protein probes to visualize sphingomyelin and ceramide phosphoethanolamine. Chem. Phys. Lipids.

[10] Panevska A., Skočaj M., Modic Š., Razinger J., Sepčić K. (2021). Aegerolysins from the fungal genus *Pleurotus*—Bioinsecticidal proteins with multiple potential applications. J. Invertebr. Pathol..

[11] Grundner M., Panevska A., Sepčić K., Skočaj M. (2021). What can mushroom proteins teach us about lipid rafts?. Membranes.

[12] MycoCosm—The Fungal Genomics Resouce (DOE Joint Genome Institute). https://mycocosm.jgi.doe.gov/mycocosm/home.

[13] Ensembl Bacteria Release 54—Jul 2022 © EMBL-EBI. http://bacteria.ensembl.org/index.html.

[14] Jumper J., Evans R., Pritzel A., Green T., Figurnov M., Ronneberger O., Tunyasuvunakool K., Bates R., Žídek A., Potapenko A. (2021). Highly accurate protein structure prediction with AlphaFold. Nature.

[15] Lee C.-H., Chang H.-W., Yang C.-T., Wali N., Shie J.-J., Hsueh Y.-P. (2020). Sensory cilia as the Achilles heel of nematodes when attacked by carnivorous mushrooms. Proc. Natl. Acad. Sci. USA.

[16] Zervakis G.I., Ntougias S., Gargano M.L., Besi M.I., Polemis E., Typas M.A., Venturella G. (2014). A reappraisal of the *Pleurotus eryngii* complex—New species and taxonomic combinations based on the application of a polyphasic approach, and an identification key to *Pleurotus* taxa associated with *Apiaceae* plants. Fungal Biol..

[17] Gupta D.K., Rühl M., Mishra B., Kleofas V., Hofrichter M., Herzog R., Pecyna M.J., Sharma R., Kellner H., Hennicke F. (2018). The genome sequence of the commercially cultivated mushroom *Agrocybe aegerita* reveals a conserved repertoire of fruiting-related genes and a versatile suite of biopolymer-degrading enzymes. BMC Genom..

[18] Meinhardt L.W., Rincones J., Bailey B.A., Aime M.C., Griffith G.W., Zhang D., Pereira G.A.G. (2008). *Moniliophthora perniciosa*, the causal agent of witches’ broom disease of cacao: What’s new from this old foe?. Mol. Plant Pathol..

[19] Fung S.-Y., Tan C.-S. (2019). Tiger milk mushroom (the *Lignosus* trinity) in Malaysia: A medicinal treasure trove. Medicinal Mushrooms.

[20] de Vries R.P., Riley R., Wiebenga A., Aguilar-Osorio G., Amillis S., Uchima C.A., Anderluh G., Asadollahi M., Askin M., Barry K. (2017). Comparative genomics reveals high biological diversity and specific adaptations in the industrially and medically important fungal genus *Aspergillus*. Genome Biol..

[21] Abdel-Azeem A.M., Salem F.M., Abdel-Azeem M.A., Nafady N.A., Mohesien M.T., Soliman E.A. (2016). Biodiversity of the genus *Aspergillus* in different habitats. New and Future Developments in Microbial Biotechnology and Bioengineering.

[22] Nierman W.C., Pain A., Anderson M.J., Wortman J.R., Kim H.S., Arroyo J., Berriman M., Abe K., Archer D.B., Bermejo C. (2005). Genomic sequence of the pathogenic and allergenic filamentous fungus *Aspergillus fumigatus*. Nature.

[23] Mullins J., Harvey R., Seaton A. (1976). Sources and incidence of airborne *Aspergillus fumigatus* (Fres). Clin. Exp. Allergy.

[24] Schuster E., Dunn-Coleman N., Frisvad J.C., Van Dijjck P. (2002). On the safety of *Aspergillus niger*—A review. Appl. Microbiol. Biotechnol..

[25] Perrone G., Susca A., Cozzi G., Ehrlich K., Varga J., Frisvad J.C., Meijer M., Noonim P., Mahakarnchanakul W., Samson R.A. (2007). Biodiversity of *Aspergillus* species in some important agricultural products. Stud. Mycol..

[26] Joint FAO/WHO Expert Committee on Food Additives (2001). Safety Evaluation of Certain Mycotoxins in Food/Prepared by the Fifty-Sixth Meeting of the Joint FAO/WHO Expert Committee on Food Additives (JECFA).

[27] Pel H.J., de Winde J.H., Archer D.B., Dyer P.S., Hofmann G., Schaap P.J., Turner G., de Vries R.P., Albang R., Albermann K. (2007). Genome sequencing and analysis of the versatile cell factory *Aspergillus niger* CBS 513.88. Nat. Biotechnol..

[28] Nayak A.P., Blachere F.M., Hettick J.M., Lukomski S., Schmechel D., Beezhold D.H. (2011). Characterization of recombinant terrelysin, a hemolysin of *Aspergillus terreus*. Mycopathologia.

[29] Machida M., Yamada O., Gomi K. (2008). Genomics of *Aspergillus oryzae*: Learning from the history of koji mold and exploration of its future. DNA Res..

[30] Vega F.E., Goettel M.S., Blackwell M., Chandler D., Jackson M.A., Keller S., Koike M., Maniania N.K., Monzón A., Ownley B.H. (2009). Fungal entomopathogens: New insights on their ecology. Fungal Ecol..

[31] Rasool S., Vidkjær N.H., Hooshmand K., Jensen B., Fomsgaard I.S., Meyling N.V. (2021). Seed inoculations with entomopathogenic fungi affect aphid populations coinciding with modulation of plant secondary metabolite profiles across plant families. New Phytol..

[32] Druzhinina I.S., Kubicek C.P. (2013). Ecological Genomics of Trichoderma. The Ecological Genomics of Fungi.

[33] Druzhinina I.S., Seidl-Seiboth V., Herrera-Estrella A., Horwitz B.A., Kenerley C.M., Monte E., Mukherjee P.K., Zeilinger S., Grigoriev I.V., Kubicek C.P. (2011). Trichoderma: The genomics of opportunistic success. Nat. Rev. Microbiol..

[34] Atanasova L., Le Crom S.L., Gruber S., Coulpier F., Seidl-Seiboth V., Kubicek C.P., Druzhinina I.S. (2013). Comparative transcriptomics reveals different strategies of Trichodermamycoparasitism. BMC Genom..

[35] Atanasova L. (2014). Ecophysiology of Trichoderma in Genomic Perspective. Biotechnology and Biology of Trichoderma.

[36] Roberts R.G., Bischoff J.F., Reymond S.T. (2011). Differential gene expression in *Alternaria gaisen* exposed to dark and light. Mycol. Prog..

[37] Simmons E.G., Roberts R.G. (1993). Alternaria themes and variations (73). Mycotaxon.

[38] Argôlo-Filho R., Loguercio L. (2013). *Bacillus thuringiensis* is an environmental pathogen and host-specificity has developed as an adaptation to human-generated ecological niches. Insects.

[39] Palma L., Muñoz D., Berry C., Murillo J., Caballero P. (2014). *Bacillus thuringiensis* toxins: An overview of their biocidal activity. Toxins.

[40] Qureshi N., Chawla S., Likitvivatanavong S., Lee H.L., Gill S.S. (2014). The Cry Toxin operon of *Clostridium bifermentans* subsp. *malaysia* is highly toxic to *Aedes* larval mosquitoes. Appl. Environ. Microbiol..

[41] Ju S., Lin J., Zheng J., Wang S., Zhou H., Sun M. (2016). *Alcaligenes faecalis* ZD02, a Novel Nematicidal Bacterium with an Extracellular Serine Protease Virulence Factor. Appl. Environ. Microbiol..

[42] Finlay B.B. (1999). Bacterial Disease in Diverse Hosts. Cell.

[43] Chieda Y., Iiyama K., Yasunaga-Aoki C., Lee J.M., Kusakabe T., Shimizu S. (2005). Pathogenicity of gacA mutant of *Pseudomonas aeruginosa* PA01 in the silkworm, *Bombyx mori*. FEMS Microbiol. Lett..

[44] Sousa A., Pereira M. (2014). *Pseudomonas aeruginosa* diversification during infection development in cystic fibrosis lungs—A review. Pathogens.

[45] Starkey M., Rahme L.G. (2009). Modeling *Pseudomonas aeruginosa* pathogenesis in plant hosts. Nat. Protoc..

[46] Bano N., Musarrat J. (2003). Characterization of a new *Pseudomonas aeruginosa* strain NJ-15 as a potential biocontrol agent. Curr. Microbiol..

[47] Specht A., Sosa-Gómez D.R., Roque-Specht V.F., Valduga E., Gonzatti F., Schuh S.M., Carneiro E. (2019). Biotic potential and life tables of *Chrysodeixis includens* (Lepidoptera: Noctuidae), *Rachiplusia nu*, and *Trichoplusia ni* on soybean and forage turnip. J. Insect Sci..

[48] Zhang S., Clark K.D., Strand M.R. (2011). The protein P23 identifies capsule-forming plasmatocytes in the moth *Pseudoplusia includens*. Dev. Comp. Immunol..

[49] Cheng X.-W., Wang L., Carner G.R., Arif B.M. (2005). Characterization of three ascovirus isolates from cotton insects. J. Invertebr. Pathol..

[50] Wang L., Xue J., Seaborn C.P., Arif B.M., Cheng X.-W. (2006). Sequence and organization of the Trichoplusia ni ascovirus 2c (*Ascoviridae*) genome. Virology.

[51] Stasiak K., Renault S., Federici B.A., Bigot Y. (2005). Characteristics of pathogenic and mutualistic relationships of ascoviruses in field populations of parasitoid wasps. J. Insect Physiol..

[52] Kraševec N., Panevska A., Lemež Š., Razinger J., Sepčić K., Anderluh G., Podobnik M. (2021). Lipid-Binding aegerolysin from biocontrol fungus *Beauveria bassiana*. Toxins.

[53] Alfaro M., Castanera R., Lavín J.L., Grigoriev I.V., Oguiza J.A., Ramírez L., Pisabarro A.G. (2016). Comparative and transcriptional analysis of the predicted secretome in the lignocellulose-degrading basidiomycete fungus *Pleurotus ostreatus*. Environ. Microbiol..

[54] Riley R., Salamov A.A., Brown D.W., Nagy L.G., Floudas D., Held B.W., Levasseur A., Lombard V., Morin E., Otillar R. (2014). Extensive sampling of basidiomycete genomes demonstrates inadequacy of the white-rot/brown-rot paradigm for wood decay fungi. Proc. Natl. Acad. Sci. USA.

[55] Castanera R., López-Varas L., Borgognone A., LaButti K., Lapidus A., Schmutz J., Grimwood J., Pérez G., Pisabarro A.G., Grigoriev I.V. (2016). Transposable elements versus the fungal genome: Impact on whole-genome architecture and transcriptional profiles. PLoS Genet..

[56] Lakkireddy K.K.R., Navarro-González M., Velagapudi R., Kües U., Savoie J.-M., Foulongne-Oriol M., Largeteau M., Barroso G. (2011). Proteins expressed during hyphal aggregation for fruting body formation in basidiomycetes. Proceedings of the 7th International Conference on Mushroom Biology and Mushroom Products.

[57] Bernheimer A.W., Avigad L.S. (1979). A cytolytic protein from the edible mushroom, Pleurotus ostreatus. Biochim. Biophys. Acta Gen. Subj..

[58] Lee S.-H., Kim B.-G., Kim K.-J., Lee J.-S., Yun D.-W., Hahn J.-H., Kim G.-H., Lee K.-H., Suh D.-S., Kwon S.-T. (2002). Comparative analysis of sequences expressed during the liquid-cultured mycelia and fruit body stages of *Pleurotus ostreatus*. Fungal Genet. Biol..

[59] Tomita T., Noguchi K., Mimuro H., Ukaji F., Ito K., Sugawara-Tomita N., Hashimoto Y. (2004). Pleurotolysin, a novel sphingomyelin-specific two-component cytolysin from the edible mushroom *Pleurotus ostreatus*, assembles into a transmembrane pore complex. J. Biol. Chem..

[60] Lukoyanova N., Kondos S.C., Farabella I., Law R.H.P., Reboul C.F., Caradoc-Davies T.T., Spicer B.A., Kleifeld O., Traore D.A.K., Ekkel S.M. (2015). Conformational changes during pore formation by the perforin-related protein pleurotolysin. PLoS Biol..

[61] Huang G., Voorspoels A., Versloot R.C.A., Van Der Heide N.J., Carlon E., Willems K., Maglia G. (2022). PlyAB nanopores detect single amino acid differences in folded haemoglobin from blood. Angew. Chem. Int. Ed..

[62] The Molecular Graphics System PyMOL (Schrödinger, LLC). https://pymol.org/2/.

[63] Berne S., Križaj I., Pohleven F., Turk T., Maček P., Sepčić K. (2002). Pleurotus and *Agrocybe* hemolysins, new proteins hypothetically involved in fungal fruiting. Biochim. Biophys. Acta.

[64] Maličev E., Chowdhury H.H., Maček P., Sepčić K. (2007). Effect of ostreolysin, an Asp-hemolysin isoform, on human chondrocytes and osteoblasts, and possible role of Asp-hemolysin in pathogenesis. Med. Mycol..

[65] Sepčić K., Berne S., Rebolj K., Batista U., Plemenitaš A., Šentjurc M., Maček P. (2004). Ostreolysin, a pore-forming protein from the oyster mushroom, interacts specifically with membrane cholesterol-rich lipid domains. FEBS Lett..

[66] Vidic I., Berne S., Drobne D., Maček P., Frangež R., Turk T., Štrus J., Sepčić K. (2005). Temporal and spatial expression of ostreolysin during development of the oyster mushroom (*Pleurotus ostreatus*). Mycol. Res..

[67] Rebolj K., Sepčić K. (2008). Ostreolysin, a cytolytic protein from culinary-medicinal oyster mushroom *Pleurotus ostreatus* (Jacq.: Fr.) P. Kumm. (Agaricomycetideae), and its potential use in medicine and biotechnology. Int. J. Med. Mushrooms.

[68] Rebolj K., Bakrač B., Garvas M., Ota K., Šentjurc M., Potrich C., Coraiola M., Tomazzolli R., Serra M.D., Maček P. (2010). EPR and FTIR studies reveal the importance of highly ordered sterol-enriched membrane domains for ostreolysin activity. Biochim. Biophys. Acta Biomembr..

[69] Rebolj K., Ulrih N.P., Maček P., Sepčić K. (2006). Steroid structural requirements for interaction of ostreolysin, a lipid-raft binding cytolysin, with lipid monolayers and bilayers. Biochim. Biophys. Acta Biomembr..

[70] Chowdhury H.H., Rebolj K., Kreft M., Zorec R., Maček P., Sepčić K. (2008). Lysophospholipids prevent binding of a cytolytic protein ostreolysin to cholesterol-enriched membrane domains. Toxicon.

[71] Sepčić K., Berne S., Potrich C., Turk T., Maček P., Menestrina G. (2003). Interaction of ostreolysin, a cytolytic protein from the edible mushroom *Pleurotus ostreatus*, with lipid membranes and modulation by lysophospholipids. Eur. J. Biochem..

[72] Ota K., Leonardi A., Mikelj M., Skočaj M., Wohlschlager T., Künzler M., Aebi M., Narat M., Križaj I., Anderluh G. (2013). Membrane cholesterol and sphingomyelin, and ostreolysin A are obligatory for pore-formation by a MACPF/CDC-like pore-forming protein, pleurotolysin B. Biochimie.

[73] Endapally S., Frias D., Grzemska M., Gay A., Tomchick D.R., Radhakrishnan A. (2019). Molecular discrimination between two conformations of sphingomyelin in plasma membranes. Cell.

[74] Skočaj M., Resnik N., Grundner M., Ota K., Rojko N., Hodnik V., Anderluh G., Sobota A.A., Maček P., Veranič P. (2014). Tracking cholesterol/sphingomyelin-rich membrane domains with the ostreolysin A-mCherry protein. PLoS ONE.

[75] Skočaj M., Yu Y., Grundner M., Resnik N., Bedina Zavec A., Leonardi A., Križaj I., Guella G., Maček P., Kreft Erdani M. (2016). Characterisation of plasmalemmal shedding of vesicles induced by the cholesterol/sphingomyelin binding protein, ostreolysin A-mCherry. Biochim. Biophys. Acta Biomembr..

[76] Resnik N., Repnik U., Kreft M.E., Sepčić K., Maček P., Turk B., Veranič P. (2015). Highly selective anti-cancer activity of cholesterol-interacting agents methyl-β-cyclodextrin and ostreolysin A/pleurotolysin B protein complex on urothelial cancer cells. PLoS ONE.

[77] Panevska A., Skočaj M., Križaj I., Maček P., Sepčić K. (2019). Ceramide phosphoethanolamine, an enigmatic cellular membrane sphingolipid. Biochim. Biophys. Acta Biomembr..

[78] Milijaš Jotić M., Panevska A., Iacovache I., Kostanjšek R., Mravinec M., Skočaj M., Zuber B., Pavšič A., Razinger J., Modic Š. (2021). Dissecting out the molecular mechanism of insecticidal activity of ostreolysin A6/pleurotolysin B complexes on western corn rootworm. Toxins.

[79] Panevska A., Hodnik V., Skočaj M., Novak M., Modic Š., Pavlic I., Podržaj S., Zarić M., Resnik N., Maček P. (2019). Pore-forming protein complexes from *Pleurotus mushrooms* kill western corn rootworm and Colorado potato beetle through targeting membrane ceramide phosphoethanolamine. Sci. Rep..

[80] Bhat H.B., Ishitsuka R., Inaba T., Murate M., Abe M., Makino A., Kohyama-Koganeya A., Nagao K., Kurahashi A., Kishimoto T. (2015). Evaluation of aegerolysins as novel tools to detect and visualize ceramide phosphoethanolamine, a major sphingolipid in invertebrates. FASEB J..

[81] Novak M., Krpan T., Panevska A., Shewell L.K., Day C.J., Jennings M.P., Guella G., Sepčić K. (2020). Binding specificity of ostreolysin A6 towards Sf9 insect cell lipids. Biochim. Biophys. Acta Biomembr..

[82] Panevska A., Glavan G., Jemec Kokalj A., Kukuljan V., Trobec T., Žužek M.C.M.C., Vrecl M., Drobne D., Frangež R., Sepčić K. (2021). Effects of bioinsecticidal aegerolysin-based cytolytic complexes on non-target organisms. Toxins.

[83] Landi N., Grundner M., Ragucci S., Pavšič M., Mravinec M., Pedone P.V., Sepčić K., Di Maro A. (2022). Characterization and cytotoxic activity of ribotoxin-like proteins from the edible mushroom *Pleurotus eryngii*. Food Chem..

[84] Juntes P., Rebolj K., Sepčić K., Maček P., Cecilija Žužek M., Cestnik V., Frangež R. (2009). Ostreolysin induces sustained contraction of porcine coronary arteries and endothelial dysfunction in middle- and large-sized vessels. Toxicon.

[85] Rebolj K., Batista U., Sepčić K., Cestnik V., Maček P., Frangež R. (2007). Ostreolysin affects rat aorta ring tension and endothelial cell viability in vitro. Toxicon.

[86] Žužek M.C., Maček P., Sepčić K., Cestnik V., Frangež R. (2006). Toxic and lethal effects of ostreolysin, a cytolytic protein from edible oyster mushroom (*Pleurotus ostreatus*), in rodents. Toxicon.

[87] Balbi T., Trenti F., Panevska A., Bajc G., Guella G., Ciacci C., Canonico B., Canesi L., Sepčić K. (2022). Ceramide aminoethylphosphonate as a new molecular target for pore-forming aegerolysin-based protein complexes. Front. Mol. Biosci..

[88] Nimri L., Spivak O., Tal D., Schälling D., Peri I., Graeve L., Salame T.M., Yarden O., Hadar Y., Schwartz B. (2017). A recombinant fungal compound induces anti-proliferative and pro-apoptotic effects on colon cancer cells. Oncotarget.

[89] Oren T., Nimri L., Yehuda-Shnaidman E., Staikin K., Hadar Y., Friedler A., Amartely H., Slutzki M., Di Pizio A., Niv M.Y. (2017). Recombinant ostreolysin induces brown fat-like phenotype in HIB-1B cells. Mol. Nutr. Food Res..

[90] Nimri L., Staikin K., Peri I., Yehuda-Shnaidman E., Schwartz B. (2018). Ostreolysin induces browning of adipocytes and ameliorates hepatic steatosis. J. Gastroenterol. Hepatol..

[91] Novak M., Čepin U., Hodnik V., Narat M., Jamnik M., Kraševec N., Sepčić K., Anderluh G. (2019). Functional studies of aegerolysin and MACPF-like proteins in *Aspergillus niger*. Mol. Microbiol..

[92] Kraševec N., Novak M., Barat S., Skočaj M., Sepčić K., Anderluh G. (2020). Unconventional secretion of nigerolysins A from *Aspergillus* species. Microorganisms.

[93] Ngai P.H.K., Ng T.B.B. (2006). A hemolysin from the mushroom *Pleurotus eryngii*. Appl. Microbiol. Biotechnol..

[94] Bhat H.B., Kishimoto T., Abe M., Makino A., Inaba T., Murate M., Dohmae N., Kurahashi A., Nishibori K., Fujimori F. (2013). Binding of a pleurotolysin ortholog from *Pleurotus eryngii* to sphingomyelin and cholesterol-rich membrane domains. J. Lipid Res..

[95] Kurahashi A., Sato M., Kobayashi T., Nishibori K., Fujimori F. (2014). Homologous genes, Pe.pleurotolysin A and Pe.ostreolysin, are both specifically and highly expressed in primordia and young fruiting bodies of *Pleurotus eryngii*. Mycoscience.

[96] Shibata T., Kudou M., Hoshi Y., Kudo A., Nanashima N., Miyairi K. (2010). Isolation and characterization of a novel two-component hemolysin, erylysin A and B, from an edible mushroom, *Pleurotus eryngii*. Toxicon.

[97] Resnik N., Sepčić K., Plemenitaš A., Windoffer R., Leube R., Veranič P. (2011). Desmosome assembly and cell-cell adhesion are membrane raft-dependent processes. J. Biol. Chem..

[98] Grundner M., Munjaković H., Tori T., Sepčić K., Gašperšič R., Oblak Č., Seme K., Guella G., Trenti F., Skočaj M. (2022). Ceramide phosphoethanolamine as a possible marker of periodontal disease. Membranes.

[99] Sakihara T., Takiguchi N., Uzawa H., Serizawa R., Kobayashi T. (2021). Erylysin A inhibits cytokinesis in *Escherichia coli* by binding with cardiolipin. J. Biochem..

[100] Fernandez Espinar M.T., Labarere J., Labarère J. (1997). Cloning and sequencing of the Aa-Pri1 gene specifically expressed during fruiting initiation in the edible mushroom *Agrocybe aegerita*, and analysis of the predicted amino-acid sequence. Curr. Genet..

[101] Pires A.B.L., Gramacho K.P., Silva D.C., Góes-Neto A., Silva M.M., Muniz-Sobrinho J.S., Porto R.F., Villela-Dias C., Brendel M., Cascardo J.C.M. (2009). Early development of *Moniliophthora perniciosa* basidiomata and developmentally regulated genes. BMC Microbiol..

[102] Berne S., Pohleven J., Vidic I., Rebolj K., Pohleven F., Turk T., Maček P., Sonnenberg A., Sepčić K. (2007). Ostreolysin enhances fruiting initiation in the oyster mushroom (*Pleurotus ostreatus*). Mycol. Res..

[103] Kües U., Liu Y. (2000). Fruiting body production in basidiomycetes. Appl. Microbiol. Biotechnol..

[104] Berne S., Sepčić K., Anderluh G., Turk T., Maček P., Poklar Ulrih N. (2005). Effect of pH on the pore forming activity and conformational stability of ostreolysin, a lipid raft-binding protein from the edible mushroom *Pleurotus ostreatus*. Biochemistry.

[105] Yap H.-Y.Y., Fung S.-Y., Ng S.-T., Tan C.-S., Tan N.-H. (2015). Genome-based proteomic analysis of *Lignosus rhinocerotis* (Cooke) Ryvarden sclerotium. Int. J. Med. Sci..

[106] Yap H.-Y.Y., Chooi Y.-H., Firdaus-Raih M., Fung S.-Y., Ng S.-T., Tan C.-S., Tan N.-H. (2014). The genome of the tiger milk mushroom, *Lignosus rhinocerotis*, provides insights into the genetic basis of its medicinal properties. BMC Genom..

[107] Iwata K., Matsuda A., Wakabayashi K., Fununaga N., Fukunaga N. (1962). Endotoxin-like substance from *Aspergillus fumigatus*. Jpn. J. Med. Mycol..

[108] Sakaguchi O., Shimada H., Yokota K. (1975). Proceedings: Purification and characteristics of hemolytic toxin from *Aspergillus fumigatus*. Jpn. J. Med. Sci. Biol..

[109] Ebina K., Yokota K., Sakaguchi O. (1982). Studies on toxin of *Aspergillus fumigatus*. XIV. Relationship between Asp-hemolysin and experimental infection for mice. Jpn. J. Med. Mycol..

[110] Ebina K., Sakagami H., Yokota K., Kondo H. (1994). Cloning and nucleotide sequence of cDNA encoding Asp-hemolysin from *Aspergillus fumigatus*. Biochim. Biophys. Acta.

[111] Kudo Y., Fukuchi Y., Kumagai T., Ebina K., Yokota K. (2001). Oxidized low-density lipoprotein-binding specificity of Asp-hemolysin from *Aspergillus fumigatus*. Biochim. Biophys. Acta Gen. Subj..

[112] Kumagai T., Nagata T., Kudo Y., Fukuchi Y., Ebina K., Yokota K. (1999). Cytotoxic activity and cytokine gene induction of Asp-hemolysin to murine macrophages. Jpn. J. Med. Mycol..

[113] Kumagai T., Nagata T., Kudo Y., Fukuchi Y., Ebina K., Yokota K. (2001). Cytotoxic activity and cytokine gene induction of Asp-hemolysin to vascular endothelial cells. J. Pharm. Soc. Jpn..

[114] Wartenberg D., Lapp K., Jacobsen I.D., Dahse H.-M., Kniemeyer O., Heinekamp T., Brakhage A.A. (2011). Secretome analysis of *Aspergillus fumigatus* reveals Asp-hemolysin as a major secreted protein. Int. J. Med. Microbiol..

[115] Rementeria A., López-Molina N., Ludwig A., Vivanco A.B., Bikandi J., Pontón J., Garaizar J. (2005). Genes y moléculas implicados en la virulencia de *Aspergillus fumigatus*. Rev. Iberoam. Micol..

[116] Nayak A.P., Green B.J., Janotka E., Hettick J.M., Friend S., Vesper S.J., Schmechel D., Beezhold D.H. (2011). Monoclonal antibodies to hyphal exoantigens derived from the opportunistic pathogen *Aspergillus terreus*. Clin. Vaccine Immunol..

[117] Nayak A.P., Green B.J., Friend S., Beezhold D.H. (2012). Development of monoclonal antibodies to recombinant terrelysin and characterization of expression in *Aspergillus terreus*. J. Med. Microbiol..

[118] Bando H., Hisada H., Ishida H., Hata Y., Katakura Y., Kondo A. (2011). Isolation of a novel promoter for efficient protein expression by *Aspergillus oryzae* in solid-state culture. Appl. Microbiol. Biotechnol..

[119] Hisada H., Tsutsumi H., Ishida H., Hata Y. (2013). High production of llama variable heavy-chain antibody fragment (VHH) fused to various reader proteins by *Aspergillus oryzae*. Appl. Microbiol. Biotechnol..

[120] Yamada R., Yoshie T., Wakai S., Asai-Nakashima N., Okazaki F., Ogino C., Hisada H., Tsutsumi H., Hata Y., Kondo A. (2014). *Aspergillus oryzae*-based cell factory for direct kojic acid production from cellulose. Microb. Cell Fact..

[121] Zaitseva J., Vaknin D., Krebs C., Doroghazi J., Milam S.L., Balasubramanian D., Duck N.B., Freigang J. (2019). Structure–function characterization of an insecticidal protein GNIP1Aa, a member of an MACPF and β-tripod families. Proc. Natl. Acad. Sci. USA.

[122] Rosado C.J., Buckle A.M., Law R.H.P., Butcher R.E., Kan W.-T., Bird C.H., Ung K., Browne K.A., Baran K., Bashtannyk-Puhalovich T.A. (2007). A common fold mediates vertebrate defense and bacterial attack. Science.

[123] Pang S.S., Bayly-Jones C., Radjainia M., Spicer B.A., Law R.H.P., Hodel A.W., Parsons E.S., Ekkel S.M., Conroy P.J., Ramm G. (2019). The cryo-EM structure of the acid activatable pore-forming immune effector Macrophage-expressed gene 1. Nat. Commun..

[124] Ni T., Jiao F., Yu X., Aden S., Ginger L., Williams S.I., Bai F., Pražák V., Karia D., Stansfeld P. (2020). Structure and mechanism of bactericidal mammalian perforin-2, an ancient agent of innate immunity. Sci. Adv..

[125] Jiao F., Dehez F., Ni T., Yu X., Dittman J.S., Gilbert R., Chipot C., Scheuring S. (2022). Perforin-2 clockwise hand-over-hand pre-pore to pore transition mechanism. Nat. Commun..

[126] Spicer B.A., Law R.H.P., Caradoc-Davies T.T., Ekkel S.M., Bayly-Jones C., Pang S.-S., Conroy P.J., Ramm G., Radjainia M., Venugopal H. (2018). The first transmembrane region of complement component-9 acts as a brake on its self-assembly. Nat. Commun..

[127] Lovelace L.L., Cooper C.L., Sodetz J.M., Lebioda L. (2011). Structure of human C8 protein provides mechanistic insight into membrane pore formation by complement. J. Biol. Chem..

[128] Dubey M., Jensen D.F., Karlsson M. (2021). Functional characterization of the AGL1 aegerolysin in the mycoparasitic fungus *Trichoderma atroviride* reveals a role in conidiation and antagonism. Mol. Genet. Genom..

[129] Pigott C.R., Ellar D.J. (2007). Role of receptors in *Bacillus thuringiensis* crystal toxin activity. Microbiol. Mol. Biol. Rev..

[130] Kelker M.S., Berry C., Evans S.L., Pai R., McCaskill D.G., Wang N.X., Russell J.C., Baker M.D., Yang C., Pflugrath J.W. (2014). Structural and biophysical characterization of *Bacillus thuringiensis* insecticidal proteins Cry34Ab1 and Cry35Ab1. PLoS ONE.

[131] Humphreys M.J., Berry C. (1998). Variants of theBacillus sphaericus binary toxins: Implications for differential toxicity of strains. J. Invertebr. Pathol..

[132] Narva K.E., Wang N.X., Herman R. (2017). Safety considerations derived from Cry34Ab1/Cry35Ab1 structure and function. J. Invertebr. Pathol..

[133] Masson L., Schwab G., Mazza A., Brousseau R., Potvin L., Schwartz J.-L. (2004). A novel *Bacillus thuringiensis* (PS149B1) containing a Cry34Ab1/Cry35Ab1 binary toxin specific for the eestern corn rootworm *Diabrotica virgifera* virgifera LeConte forms ion channels in lipid membranes. Biochemistry.

[134] Moellenbeck D.J., Peters M.L., Bing J.W., Rouse J.R., Higgins L.S., Sims L., Nevshemal T., Marshall L., Ellis R.T., Bystrak P.G. (2001). Insecticidal proteins from *Bacillus thuringiensis* protect corn from corn rootworms. Nat. Biotechnol..

[135] Li H., Olson M., Lin G., Hey T., Tan S.Y., Narva K.E. (2013). *Bacillus thuringiensis* Cry34Ab1/Cry35Ab1 interactions with western corn rootworm midgut membrane binding sites. PLoS ONE.

[136] Wang H., Eyun S., Arora K., Tan S., Gandra P., Moriyama E., Khajuria C., Jurzenski J., Li H., Donahue M. (2017). Patterns of gene expression in western corn rootworm (*Diabrotica virgifera virgifera*) neonates, challenged with Cry34Ab1, Cry35Ab1 and Cry34/35Ab1, based on next-generation sequencing. Toxins.

[137] Schnepf H.E., Lee S., Dojillo J.A., Burmeister P., Fencil K., Morera L., Nygaard L., Narva K.E., Wolt J.D. (2005). Characterization of Cry34/Cry35 binary insecticidal proteins from diverse *Bacillus thuringiensis* strain collections. Appl. Environ. Microbiol..

[138] Berry C., O’Neil S., Ben-Dov E., Jones A.F., Murphy L., Quail M.A., Holden M.T.G., Harris D., Zaritsky A., Parkhill J. (2002). Complete sequence and organization of pBtoxis, the toxin-coding plasmid of *Bacillus thuringiensis* subsp. israelensis. Appl. Environ. Microbiol..

[139] Gillis A., Fayad N., Makart L., Bolotin A., Sorokin A., Kallassy M., Mahillon J. (2018). Role of plasmid plasticity and mobile genetic elements in the entomopathogen *Bacillus thuringiensis* serovar israelensis. FEMS Microbiol. Rev..

[140] Barloy F., Lecadet M.M., Delécluse A. (1998). Cloning and sequencing of three new putative toxin genes from *Clostridium bifermentans* CH18. Gene.

[141] Juárez-Pérez V., Delécluse A. (2001). The Cry toxins and the putative hemolysins of *Clostridium bifermentans* ser. malaysia are not involved in mosquitocidal activity. J. Invertebr. Pathol..

[142] Barloy F., Delécluse A., Nicolas L., Lecadet M.M. (1996). Cloning and expression of the first anaerobic toxin gene from *Clostridium bifermentans* subsp. malaysia, encoding a new mosquitocidal protein with homologies to *Bacillus thuringiensis* delta-endotoxins. J. Bacteriol..

[143] Yalpani N., Altier D., Barry J., Kassa A., Nowatzki T.M., Sethi A., Zhao J.-Z., Diehn S., Crane V., Sandahl G. (2017). An Alcaligenes strain emulates *Bacillus thuringiensis* producing a binary protein that kills corn rootworm through a mechanism similar to Cry34Ab1/Cry35Ab1. Sci. Rep..

[144] Pérez Ortega C., Leininger C., Barry J., Poland B., Yalpani N., Altier D., Nelson M.E., Lu A.L. (2021). Coordinated binding of a two-component insecticidal protein from *Alcaligenes faecalis* to western corn rootworm midgut tissue. J. Invertebr. Pathol..

[145] Miklavič Š., Kogovšek P., Hodnik V., Korošec J., Kladnik A., Anderluh G., Gutierrez-Aguirre I., Maček P., Butala M., Miklavič S. (2015). The *Pseudomonas aeruginosa* RhlR-controlled aegerolysin RahU is a low-affinity rhamnolipid-binding protein. FEMS Microbiol. Lett..

[146] Rao J., Elliott M.R., Leitinger N., Jensen R.V., Goldberg J.B., Amin A.R. (2011). RahU: An inducible and functionally pleiotropic protein in *Pseudomonas aeruginosa* modulates innate immunity and inflammation in host cells. Cell. Immunol..

[147] Rao J., DiGiandomenico A., Unger J., Bao Y., Polanowska-Grabowska R.K.R.K., Goldberg J.B.J.B. (2008). A novel oxidized low-density lipoprotein-binding protein from *Pseudomonas aeruginosa*. Microbiology.

[148] Kočar E., Lenarčič T., Hodnik V., Panevska A., Huang Y., Bajc G., Kostanjšek R., Naren A.P.A.P., Maček P., Anderluh G. (2021). Crystal structure of RahU, an aegerolysin protein from the human pathogen *Pseudomonas aeruginosa*, and its interaction with membrane ceramide phosphorylethanolamine. Sci. Rep..

[149] Cui L., Cheng X., Li L., Li J. (2007). Identification of *Trichoplusia ni* ascovirus 2c virion structural proteins. J. Gen. Virol..

[150] Liu Y.-Y., Xian W.-F., Xue J., Wei Y.-L., Cheng X.-W., Wang X. (2018). Complete genome sequence of a renamed isolate, *Trichoplusia ni* Ascovirus 6b, from the United States. Genome Announc..

[151] Huang G.-H., Hou D.-H., Wang M., Cheng X.-W., Hu Z. (2017). Genome analysis of *Heliothis virescens* ascovirus 3h isolated from China. Virol. Sin..

[152] Zaghloul H.A.H., Hice R., Arensburger P., Federici B.A. (2022). Early in vivo transcriptome of *Trichoplusia ni* ascovirus core genes. J. Gen. Virol..

[153] Merkel J.S., Regan L. (1998). Aromatic rescue of glycine in β sheets. Fold. Des..

[154] Tamura K., Stecher G., Kumar S. (2021). MEGA11: Molecular evolutionary genetics analysis version 11. Mol. Biol. Evol..

[155] Anderluh G., Kisovec M., Kraševec N., Gilbert R.J.C. (2014). Distribution of MACPF/CDC Proteins. Subcell. Biochem..

[156] Bravo A., Gill S.S., Soberón M. (2007). Mode of action of Bacillus thuringiensis Cry and Cyt toxins and their potential for insect control. Toxicon.

[157] Ruiz-Dueñas F.J., Barrasa J.M., Sánchez-García M., Camarero S., Miyauchi S., Serrano A., Linde D., Babiker R., Drula E., Ayuso-Fernández I. (2021). Genomic analysis enlightens *Agaricales* lifestyle evolution and increasing peroxidase diversity. Mol. Biol. Evol..

[158] Xiao G., Ying S.-H., Zheng P., Wang Z.-L., Zhang S., Xie X.-Q., Shang Y., St. Leger R.J., Zhao G.-P., Wang C. (2012). Genomic perspectives on the evolution of fungal entomopathogenicity in *Beauveria bassiana*. Sci. Rep..

[159] Dang H.X., Pryor B., Peever T., Lawrence C.B. (2015). The *Alternaria* genomes database: A comprehensive resource for a fungal genus comprised of saprophytes, plant pathogens, and allergenic species. BMC Genom..

[160] Folman L.B., Klein Gunnewiek P.J.A., Boddy L., De Boer W. (2008). Impact of white-rot fungi on numbers and community composition of bacteria colonizing beech wood from forest soil. FEMS Microbiol. Ecol..

[161] Kneitel J. (2008). Gause’s Competitive Exclusion Principle. Encyclopedia of Ecology.

[162] Klepzig K.D. (1998). Competition between a biological control fungus, *Ophiostoma piliferum*, and symbionts of the southern pine beetle. Mycologia.

[163] Ulyshen M.D. (2016). Wood decomposition as influenced by invertebrates. Biol. Rev..

[164] Al-Deen I.H.S., Twaij H.A.A., Al-Badr A.A., Istarabadi T.A.W. (1987). Toxicologic and histopathologic studies of *Pleurotus ostreatus* mushroom in mice. J. Ethnopharmacol..

[165] Wang C., Wang S. (2017). Insect pathogenic fungi: Genomics, molecular interactions, and genetic improvements. Annu. Rev. Entomol..

[166] Li S., Yi W., Chen S., Wang C. (2021). Empirical support for the pattern of competitive exclusion between insect parasitic fungi. J. Fungi.

[167] Zheng P., Xia Y., Zhang S., Wang C. (2013). Genetics of Cordyceps and related fungi. Appl. Microbiol. Biotechnol..

[168] Mei L., Chen M., Shang Y., Tang G., Tao Y., Zeng L., Huang B., Li Z., Zhan S., Wang C. (2020). Population genomics and evolution of a fungal pathogen after releasing exotic strains to control insect pests for 20 years. ISME J..

[169] Parsa S., Ortiz V., Vega F.E. (2013). Establishing fungal entomopathogens as endophytes: Towards endophytic biological control. J. Vis. Exp..

[170] Ownley B.H., Gwinn K.D., Vega F.E. (2009). Endophytic fungal entomopathogens with activity against plant pathogens: Ecology and evolution. The Ecology of Fungal Entomopathogens.

[171] Wong D., Bazopoulou D., Pujol N., Tavernarakis N., Ewbank J.J. (2007). Genome-wide investigation reveals pathogen-specific and shared signatures in the response of *Caenorhabditis elegans* to infection. Genome Biol..

[172] Zhang F., Peng D., Cheng C., Zhou W., Ju S., Wan D., Yu Z., Shi J., Deng Y., Wang F. (2016). *Bacillus thuringiensis* crystal protein Cry6Aa triggers *Caenorhabditis elegans* necrosis pathway mediated by aspartic protease (ASP-1). PLoS Pathog..

[173] Pestsov G.V., Lushnikov O.V., Glazunova A.V. (2019). *Nematopathogenic fungi* as the basis of the biological control of root-knot nematodes. Agrar. Sci..

[174] Soanes D., Richards T.A. (2014). Horizontal gene iransfer in eukaryotic plant pathogens. Annu. Rev. Phytopathol..

[175] Schnepf E., Crickmore N., Van Rie J., Lereclus D., Baum J., Feitelson J., Zeigler D.R., Dean D.H. (1998). *Bacillus thuringiensis* and its pesticidal crystal proteins. Microbiol. Mol. Biol. Rev..

[176] Sajid M., Geng C., Li M., Wang Y., Liu H., Zheng J., Peng D., Sun M. (2018). Whole-genome analysis of *Bacillus thuringiensis* revealing partial genes as a source of novel Cry toxins. Appl. Environ. Microbiol..

[177] Liu X., Lieberman J. (2020). Knocking ’em dead: Pore-forming proteins in immune defense. Annu. Rev. Immunol..

[178] Scopus Title-Abstract-Keywords Search. https://www.scopus.com/search/form.uri?display=basic#basic.

[179] Grigoriev I.V., Nikitin R., Haridas S., Kuo A., Ohm R., Otillar R., Riley R., Salamov A., Zhao X., Korzeniewski F. (2014). MycoCosm portal: Gearing up for 1000 fungal genomes. Nucleic Acids Res..

[180] Howe K.L., Contreras-Moreira B., De Silva N., Maslen G., Akanni W., Allen J., Alvarez-Jarreta J., Barba M., Bolser D.M., Cambell L. (2020). Ensembl Genomes 2020—enabling non-vertebrate genomic research. Nucleic Acids Res..

[181] Ensembl Fungi Release 54—Jul 2022 © EMBL-EBI. https://fungi.ensembl.org/index.html.

[182] Kubicek C.P., Herrera-Estrella A., Seidl-Seiboth V., Martinez D.A., Druzhinina I.S., Thon M., Zeilinger S., Casas-Flores S., Horwitz B.A., Mukherjee P.K. (2011). Comparative genome sequence analysis underscores mycoparasitism as the ancestral life style of *Trichoderma*. Genome Biol..

[183] Mondego J.M.C., Carazzolle M.F., Costa G.G.L., Formighieri E.F., Parizzi L.P., Rincones J., Cotomacci C., Carraro D.M., Cunha A.F., Carrer H. (2008). A genome survey of *Moniliophthora perniciosa* gives new insights into Witches’ Broom Disease of cacao. BMC Genom..

[184] Arnaud M.B., Cerqueira G.C., Inglis D.O., Skrzypek M.S., Binkley J., Chibucos M.C., Crabtree J., Howarth C., Orvis J., Shah P. (2012). The *Aspergillus* Genome Database (AspGD): Recent developments in comprehensive multispecies curation, comparative genomics and community resources. Nucleic Acids Res..

[185] Fedorova N.D., Khaldi N., Joardar V.S., Maiti R., Amedeo P., Anderson M.J., Crabtree J., Silva J.C., Badger J.H., Albarraq A. (2008). Genomic islands in the pathogenic filamentous fungus *Aspergillus fumigatus*. PLoS Genet..

[186] Ronning C.M., Fedorova N.D., Bowyer P., Coulson R., Goldman G., Stanley Kim H., Turner G., Wortman J.R., Yu J., Anderson M.J. (2005). Genomics of *Aspergillus fumigatus*. Rev. Iberoam. Micol..

[187] Joardar V., Abrams N.F., Hostetler J., Paukstelis P.J., Pakala S., Pakala S.B., Zafar N., Abolude O.O., Payne G., Andrianopoulos A. (2012). Sequencing of mitochondrial genomes of nine *Aspergillus* and *Penicillium* species identifies mobile introns and accessory genes as main sources of genome size variability. BMC Genom..

[188] Vesth T.C., Nybo J.L., Theobald S., Frisvad J.C., Larsen T.O., Nielsen K.F., Hoof J.B., Brandl J., Salamov A., Riley R. (2018). Investigation of inter- and intraspecies variation through genome sequencing of *Aspergillus* section Nigri. Nat. Genet..

[189] Andersen M.R., Salazar M.P., Schaap P.J., Van De Vondervoort P.J.I., Culley D., Thykaer J., Frisvad J.C., Nielsen K.F., Albang R., Albermann K. (2011). Comparative genomics of citric-acid-producing *Aspergillus niger* ATCC 1015 versus enzyme-producing CBS 513.88. Genome Res..

[190] Aguilar-Pontes M.V., Brandl J., McDonnell E., Strasser K., Nguyen T.T.M., Riley R., Mondo S., Salamov A., Nybo J.L., Vesth T.C. (2018). The gold-standard genome of *Aspergillus niger* NRRL 3 enables a detailed view of the diversity of sugar catabolism in fungi. Stud. Mycol..

[191] Machida M., Asai K., Sano M., Tanaka T., Kumagai T., Terai G., Kusumoto K.-I., Arima T., Akita O., Kashiwagi Y. (2005). Genome sequencing and analysis of *Aspergillus oryzae*. Nature.

[192] Stover C.K., Pham X.Q., Erwin A.L., Mizoguchi S.D., Warrener P., Hickey M.J., Brinkman F.S.L., Hufnagle W.O., Kowalik D.J., Lagrou M. (2000). Complete genome sequence of *Pseudomonas aeruginosa* PAO1, an opportunistic pathogen. Nature.

[193] ClustalW 2.1 (Kyoto University Bioinformatics Centre). https://www.genome.jp/tools-bin/clustalw.

[194] Jones D.T., Taylor W.R., Thornton J.M. (1992). The rapid generation of mutation data matrices from protein sequences. Bioinformatics.

[195] Pettersen E.F., Goddard T.D., Huang C.C., Couch G.S., Greenblatt D.M., Meng E.C., Ferrin T.E. (2004). UCSF Chimera—A visualization system for exploratory research and analysis. J. Comput. Chem..

[196] Pettersen E.F., Goddard T.D., Huang C.C., Meng E.C., Couch G.S., Croll T.I., Morris J.H., Ferrin T.E. (2021). UCSF ChimeraX: Structure visualization for researchers, educators, and developers. Protein Sci..

[197] UCSF ChimeraX. http://www.cgl.ucsf.edu/chimera/.

[198] Goddard T.D., Huang C.C., Meng E.C., Pettersen E.F., Couch G.S., Morris J.H., Ferrin T.E. (2018). UCSF ChimeraX: Meeting modern challenges in visualization and analysis. Protein Sci..

[199] Mirdita M., Schütze K., Moriwaki Y., Heo L., Ovchinnikov S., Steinegger M. (2022). ColabFold: Making protein folding accessible to all. Nat. Methods.

[200] Waterhouse A.M., Procter J.B., Martin D.M.A., Clamp M., Barton G.J. (2009). Jalview Version 2—A multiple sequence alignment editor and analysis workbench. Bioinformatics.

[201] Larkin M.A., Blackshields G., Brown N.P., Chenna R., McGettigan P.A., McWilliam H., Valentin F., Wallace I.M., Wilm A., Lopez R. (2007). Clustal W and Clustal X version 2.0. Bioinformatics.

[202] Drozdetskiy A., Cole C., Procter J., Barton G.J. (2015). JPred4: A protein secondary structure prediction server. Nucleic Acids Res..

