# Supplementary material for: Towards Understanding the Function of Aegerolysins
Source: Toxins (Basel). 2022 Sep 11;14(9):629. doi: 10.3390/toxins14090629 (PMC9505663; doi:10.3390/toxins14090629)
Supplement: Supplementary file 1 [file toxins-14-00629-s001.zip › toxins-1889997-supplementary.pdf]

Supplementary file

Towards understanding the function of aegerolysins

Nada Kraševec [1,] * and Matej Skočaj [2]

Sequences of aegerolysins and partner proteins

1. Sequences of aegerolysins

>**OlyA6_6MYJ_139**
GMAYAQWVIIIIHNVGSQDVKIKNLKASWGKLHADGDKDAEVSASNYEGKIVKPDEKLQINASGRSDAAEGTTGTFDLVDPADGDKQVRHFYWDSPWGSKTNTWTVSGS
NTKWMIEYSGQNLDSGALGTITVDTLKKGN

>**PlyA2_138**
MAYAQWVIIIIHNVGSKDVKIVNLKPSWGKLHADGDKDTEVSASKYEGTVIKPDEKLQINACGRSDAAEGTTGTFDLVDPADGDKQVRHFYWDCPWGSKANTWTVSGSN
TKWMIEYSGQNLDSGALGTITVDTLKKGN

>**EryA_138**
MAYAQWVIILIHNVGQQNVKIKNLNASWGKLYADGDKDTEVPASKYEGMVIAPDDQVQINACGREDAAEGTTGTFDLVDPNDSDKQVRHFAWDCPWGTKANSWVVGGSN
SKWMIEYTGQNLDSGALGTITVNTLRIGN

>**Aa-Pri1_145**
MDSNKDERAYAQWVIIILHNVGSSPFKIANLGLSWGKLYADGNKDKEVYPSDYNGKTVGPDEKIQINSCGRENASSGTEGSFDIVDPNDGNKTIRHFYWECPWGSKRNT
WTPSGSNTKWMVEWSGQNLDSGALGTITVDVLRKGN

>**MpPRIA1_138**
MAYAQWVVIVLHNVGNSPMKVKNVSLDWGKFHVDGNKDKEIGKDQIEGKVIGPDEKFQINSCGRSDASSGTEGSFDLVDTKDGDKTIRHCYWECPWGSKTNTWTVNGSN
SKWMVEHQGANLYGGALGTITVDAMKKGN

>**MpPRIA2_135**
MESFSDKDKAYDHWAVFILSNIGGFGRMRVENFTVTSGKLHREGDKDSDVDEDKYNGTVIGPGEKLQINACGSGSHPSGSSGKFDLLDPDEGDKLIRHFVWDCPFGSET
NTWDVSGSNTKWMVEFTGGNINGGAL

>**GME7309_149**
MSTDDPVLRAYAQWVTIRINNIGTIPIKLKNLSLSWGKLYVNGNKDNEVGTDRYEGSVISPNEWLEFSSCGRSDASSGTEGRFDLVDPGAGDKIIRNFYWDCPWGSKSN
QWSVSGSNSKFMIEHYGANYSSGALGTITVDVLNKPGVPA

>**Asp-HS_139**
MASVQAYAQWVTVHLINSMSSETLSIQNASLSWGKWYKDGDKDAEITSEDVQQKTAPPGGSVNVNSCGRSDASSGTTGGFDLYDGNTKIGRVHWDCPWGSKTNDFDVGE
RNKNYWVEIGTWNKYGGAIGTVDVEVGRKR

>**Asp-HS-like_139**
MASTTGQQFLDIEIKDDMKYDIRIENAHIESGEFYREGDQNDTLTTDDIEDMIIRHNGGLRHVCSCGEEKGFKGLQGTIDLIDDVKDAKICTLEWNAPMEPGKRNTFML
RDQDPRYHIDIGQWNESGILGKVPVTISDE

>**NigA1_142**
MPAAGPMDDQWVSFHIRDHLEKGEITVRHTVIEGGEFHDPNNRRQSLTEDEIDEITIPSYGIGEICARGRRGSEGRLDLFHDEEKICELHWDNRQGNPVNIVEMLDSNS
KYRVEHGGWSPEAGPLGHVYIDILEQQKKKNSK

>**NigA2_145**
MAERAEAQWVHIRVVNSLSFDTLSVRNTWLGWGKFHKENNKSAEIPVSDINALRAAPGGSFNVFACGRAHSPSGTEGSFDVYNGDVRVAYIYWDCPWGSKRNQFRVDRI
ADDYWVETGYWNQSGGAIGSVTVEIGRRDRAIRSSL

>**Ter_130**
MDDSQWVSIHIRDRLAQGNITIRESFLYEGQFHSPEDEKKALTEDDIDQLIIPSEGIGEVCARGRRGSEGWMDLFDGEKKICELHWDNRTKRPSNEFEVIDGDKDYKIE
CSGWSPQAGPLGHVFIDISAA

>**AoHlyA_141**
MDTDLNKYNSKWVEFHIRDHLKDGEISVRHTVIEDGEFQDPNNRRKSIHEDVIDEIVIPSDGIGEICAHGRRGSEGRLDLFHGNDKICELHWDDRDGRRENLVEMLDES
DKYRIEHGGWSPEANGPLGHVYVDVWAKDKSK

>**BlyA_137**
MAYAQWVSVTIVNIVNSGIISVQDAYVLWGKFYEGDNKNNEIQPAQVNKTTVAPGAQVTVAASGRSDSSSGVEGGFNIHLGGQKICGVYFNCPWGEKTNDFQIRDYDPA
TSPYHINTSPINRDSGALGNVTVTVLLR

>**Agl1_146**
MGDARSEDQWVSVRVLNRMRKNDISFKNAALSWGKFYSGSKSNEISAATVDETVVHTGITKSADSCGRSGAASGVEGHLDLYDGTKKICTLYWNCPWGSAVNDFQVRDY
SAVTSDYSVAVENWNRKDGPLGNVDIAVSLLGVREKR

>**L152_135**
MAYAQWVVIHIINSFRNGSISVKNAEAMWGKFHKNGNKDAEIGAGEVNKISVGAGSSEKVSSCGRSDSASGTEGKFDIYDGDNRVCTVYWSCPWGSKSNNFQIQNRNKD
YGITLGDWNQDSGALGEVDVDISRKG

>**Cry34Ab1_4JOX_118**
AREVHIDVNNKTGHTLQLEDKTKLDGGRWRTSPTNVANDQIKTFVAESNGFMTGTEGTIYYSINGEAEISLYFDNPFAGSNKYDGHSNKSQYEIITQGGSGNQSHVTYT
IQTTSSRYG

>**Cbm17.1_153**
MNNNCEVNCENTEENKSNRAYRQWVKFHIEAVNEGLKIRNASLKWGKFHDPNNKDIPISPEDISKINIEKHDTAIIASSGKENTASGTEGVFYICDENEDKIAAIYWDC
PWSGSNKLTIDKYNTKFAIEQSPTMISSSGAIGDVNLKVSKIWA

>**Cbm17.2_152**
MNNNCEVNCENTEENKSNRAYRQWVKFHIEAVHSNLKIRNASLTWGKFHDPYNKDIPISPEDISKINIGIGRIEIIASSGKENTASGTEGVFYICDEHEEKIVTVYWDC
PWSGSNKFELRDKNPNFIIETYPTMISSSGAIGDVNIRVFRRF

>**AfIP-1A_5V3S_140**
EESKIRAYAQWXEITIFVVNSNFKVEGAYLRWGKFHVPGDKDKEISPSQINGTIIKDEDSYTIASCGRENASSGTEGGFSLYDGDKLVFEYYWDCPWSGSNSDELTVKD
KENYTVIKKGGGSPSGAXGNIFITVVKKSLE

>**RahU_6ZC1_137**

AYAEWIAVKVVVSKSIGTVGIRNATLQWGKFYRYTNKDDEISTEEVSSMNVQAGSPQWIASCGRENASSGTQGSFDCYDGNTKMGTFSWDDPWKSGATNTWSFTPASAD
YAGITSGGNATGTDIGEVTLTLGRFSEN

>**P23_209**

MKTTYVVRLAALAAVLLASGKVDAGNAHYVERFERFSPEQSIGIIMCLANLVFTNNISVLSIMACVSGLSEPQPFAWGEGNYINFKIVNGESVSESGMIMNAELTWGKW
QKDGKDIDTVNRTEFDSKGKRAEFSAVGRTGVPSGAEGFFEIFEDDKVIAKVIFDVPFMGDNKLKIKQLDKGFLCDDSGFDAGGSLTIEIVCHKLGAAVP

>**TnAV2c_gp029_222**

MKGLFLITIGILAWVGGFTTNAEVVDGSCGDSNHWVPTASCQSTKKLSSVKEDWEHRLCNFFVCLTKLATSGGSFDMSEIMKCINTAIPRGENNAVQFHVHNGAGAKNN
GFISNAVLTWGKWQVNGVDVVSVNGMKFSNTNTAIFKAMGRENSPSGTEGSFDIIENDSELAATVTFSVPWSGQDKINVKNAGGSNYVCAPSETRGLVGPLSYDILCHK
INNS

2. Sequences of partner proteins

>**PlyB_4OEJA_523**

MEAVLSRQAATAEAIGRFQDSSTSVGLVAGSPSTRIRRQADNVVLKSTSQAGDTLNDVIQDPTRRNKLINDNNLLKGIIMGRDGPVPSSRELIVRPDTLRAIINNRATI
ETTTMEAEFTETLMESNYNSASVKVSAPFITANSEYSESSSFKNTETEKSMYTSSRYLFPQGRIDFTTPDSGFDDVIKLSPQFTSGVQAALAKATGTEKREALQNLFQE
YGHVFRTKVHIGGVLSAHTMETFSRSENETEVKQDVKAGLEGAVKGWGGGATAGHGNTQGTITTSQNRKLNVKYIVNGGDYTKIQNTEEWVASTNQSEHWRVIEVTEVT
AVADLLPQPIRGQVKDLLKPLLGKWVDVEKVPGLESLPVSVYRPKGAIPAGWFWLGDTADASKALLVKPTLPARSGRNPALTSLHQGSGMTEQPFVDLPQYQYLSTYFG
SFAHDTPPGSTLRGLRPDHVLPGRYEMHGDTISTAVYVTRPVDVPFPEDECFDLKSLVRVKLPGSGNPPKPRSALKKSMVLFDSGEK

>**EryB_522**

MAAVLSRQAATAEAVERFQDSSTSVGLVAGSPSRIRRQADNVVLKSISQAGDTLNDVIQDPTRRNKLINDNNLLKGIIMGRDGPVPSSRELIERPDTLRAIINNRATIE
TTTVEAEFTETLMESNYNSASVKVSAPFVTANSEYSESSSFKNTETEKSMYTSSRYLFPQGRIDFTMPDSGFDDVIKLSPQFTSGVQAALAKATGTEKREALQDLFLEY
GHVFRTKVHIGGVLSAHTMETFSRSENETEVKQDIKAGLEGAVKGWGGGATAGHGNTQGTITTSQNRKLDVKYIVNGGDYTKIQNTEEWVASTNQSEHWRVIEVTEVTA
VADLLPQPIRGQVKDLLKPLLGKWVDVEKVPGLESFPVSVYRPKDAIPAGWFWLGDTADASKALLVKPTLPARSGRNPALTSLHESSGMTEQPFVDLPQYQYLSTYFGS
FAYDTPPGSTLRGLRPDHILPGRYEMHGDTIGTAVYVTRPVDVPFPEDECFDLKSVVRVKLPGSGNPPKPRWALKKSMVLFDSGEE

>**Asp-HSB_599**

MNIFFRIWFYRKCLIVPCMQQVRCVEYHYAIAFLTIPCVKPSVGSDYDESDCLTGRDRSLSCDYYVHIRATLKECHHEFNVPLPFRFTFISITLYSFSSHRKPKMVAEL
VPYVDGMQRGQGYDTYLQELRVANAVTITSKSPPSETYDLTYKSVQIEEYTELAKSLEITAGAAISGWGQSAQIDTSYLNRSKFESATTTYQVEVSSQQQATIDNTYSF
NKISTTDPNASYGDRFVADFIKGGKFLARVSISSISKSSTEEVKEAAKVAFTMYGVTGEVTEEVKHAVSSIQKNSRITIWIHISGGGTKLGETKRIDSGPDDEDSPLFK
IKKEADNFYQELKDGKHKYRRFGVLWKYTNVPDFNNAFDPFDYSAANKKVRPSLVMRSIMEFDELCQSWNFFEDFTQYGVYIDNVKKMPVDKFLGGRQQQADLYDEGTR
VNVAISNKIAAIDKDPTDVDKPLPYPKPYEFQRKVLRALKTVTYIAQERSVDGGRLTDIALPTLQGGAEKLFEFKAFDFDAVVGTSVVSFGKRDSSYICLNGQRASDFG
YKEESVFWTFPFPVDQVAEQKINVSKLKSADLIRLSRTETGPSFLFDVYTEKSS

>**NigB1_473**

MAELVPYFDGMQQGQGYNTYLQQIGVADAVTITPGQPESATYDLFYRSERIDEYTKLAQSLEISAGAAISGWGQSSQIDAGYLDRLSFESSTVTYQVEVSSRQQASIGN
SYSFNQISTDTPNRLYGDRFIADFIRGGQYFARVSISAVNKSTSQEIRQASEVAFSMYGVTGSVTNEVRSAVETINRHSRVTVWIHTSGGGGRSGVIHAEPHDGEDSPL
FAIKEGADDFYQELREGKHRYRRFALLWRYTNVPNFNNAFTPFDYAFANQRSWYLFGDFTQYDAYLELIRKIPVQKFINGREQQAELYEEGANILSGIRTKVQAVNENP
EEINSPFSYPSPEDYKKLVLLAIRTVTMIAQERTLDNGSFSDIALPTLQGNARTLFKFQTYDFDTGFGNTVVSFGRRDSSFICLSGQRVSDGYREESVFWAFEDPVEEV
SEQQVHVASMRTRDLLLLSRTDPGPRPLFTFYAKSSW

>**BlyB_497**

MRIGVDTSVLGRFPVALLFWNVAYGETVPFSNGLTLGQGYNTYLNHGCRHNAVNVSLKPDSQSPQLQIDYRAIQINKYEQLVNVLETSAGAAVKATVESGPSAGTTASF
LDKSSFESSFLTYVVKVDVEKQTSSDASFALNWDNAADISVYCDRFISHFIKGGALFARVDIKTTDSSKHKAIEQSAKAAFNAFGANVELTQSLKTSMTEIQKSSEVDM
QFVYIGAPSDAQQTVSGNGSGGDAAQLVQLKTIADNFLARAKDSEWKRRAVLEKYEHVPNWSKQFQVPDYTTAKDYSWSVFKDSSSYQALLKGAQQISPENYIGGATTR
NQVLGVINKALSDILDWVKKVTVDPASAAKLPAVTAPGEYFETFLNSLTTTKYVAQSFETASHVSTHVIEPSLRDGATKLADAEAFGFGTVAGSIKVAFGKSLQGESYV
CLPDQPMAPGSKTISELWGMKKATGNFSKPVHVVPVPQVGVIRLSTEARAPAGSLFTFFTK

>**L152B_479**

MGEVVPYKAGMQRGQGYNTYLQALCVKDAVTIERHDDKDPAFQREYYSEFIEEYEKIAKSMRISAGAAVSGWGQEGNVNVDVLNRSEFETSTLTYEVKVLVQHQVSVVD
RHSFNKIQTDNKHATYGDRFISDFIKGGHFYARVSITAKNSSETSELKQSAEVAMTMYGVSGKITQEVESAVSSIKRNASVKITIIESTGTGKSGTSGGGYAVKAEESS
DLLAVKEKADQFYQDADTGKHSYVLLNLSNFENYFTPFDYQIASLRSWALFNDFTLYKAIETMIKAVPTSKFKDGPERKTQLSNQAINIFESIRNRVIRISEHPEEAKQ
KSDHMEPDVFRLEVLNSIQTKLFHAQSKPIPNTDDYWTDVILPSKGSGEQHLFTFPAFDFGDLIGTEVVSFGKKNNGEEYNCLIGERATSLDGYKELSHFWIFPDSVEK
FAMQMYGVSKVSTRNYMRVYAADQSDIENPRPYQRFWFFVPSA

>**AfIP-1B_703**

MDIEAKSINPLMGITVGSTVLGNDFKPSVLINPITRMITQKEIEMGMSGKSQYVYTDSLNEGTIGFSGAYGPSGIAKFTSAVAVSVSNATASEYKSIKVSYNISMISGI
EYIDFDNLTVEDVLNSLSAGPKNLSLKVLEKFIAARDCSGSSIEKDELMKEWVKSLQNFISSYGDGLVVGAIWGGMGSVSTEMTSKKTEDSWKYGETAEFSYSGIGSSV
SIAQTYNGSQKDQSSEVEVSCKALASGGCVESQVNSWFDVVANKSFAEISGISLLDKAPMQSSVSPPPKIPDFLKPEKNAEITEKLDTIKKLGDSEEFALASGYEEAKK
TNPNLTFEEFKSTVRDKNNIDGLNELASKVQENSLDVLAEGSISRRNKNLSLQGVRTISLDSSSDYAVLGAWIANWSDIFPWMSMGYMNEISDAEVAEYILKIRCMMQD
LSTLNTIYNTFNACNIKLDFCHLNSASQVADSFKIAQGVLSDNVESDDAVEIAFNSLSDEAKKIYTAWNEIGFLRNAELGLGLLIGDQSVSSEIIDVKPIPYPEVTYKA
AYCSYGNNNPTAFSSFIKMLPFIDTNGDIYAFGPSLMLLRKALPEKMIFTKGGEMAMKLTADKDTGILTNDSVKLIPIPYSAAKGIQWRGQGQGRSLASSQSLQDQFAA
LEKELGNLNICTLSSDSWSKDWTYTVPYTLRKISTTYIGTVEKINSIFG

>**Cry35Ab1_4JP0_383**

MLDTNKVYEISNHANGLYAATYLSLDDSGVSLMNKNDDDIDDYNLKWFLFPIDDDQYIITSYAANNCKVWNVNNDKINVSTYSSTNSIQKWQIKANGSSYVIQSDNGKV
LTAGTGQALGLIRLTDESSNNPNQQWNLTSVQTIQLPQKPIIDTKLKDYPKYSPTGNIDNGTSPQLMGWTLVPCIMVNDPNIDKNTQIKTTPYYILKKYQYWQRAVGSN
VALRPHEKKSYTYEWGTEIDQKTTIINTLGFQINIDSGMKFDIPEVGGGTDEIKTQLNEELKIEYSHETKIMEKYQEQSEIDNPTDQSMNSIGFLTITSLELYRYNGSE
IRIMQIQTSDNDTYNVTSYPNHQQALLLLTNHSYEEVEEITNIPKSTLKKLKKYYF

>**Cry16Aa_613**

MNTNIFSTHLEFSKGVASVFKVIDTIHNISKNNNFNNILTQDFIIDTILSILWEDPNENEIFSSMIEDGETITNKNLSAQTKEGLLLNSNSFGLKFKYYNNAFRSWIDN
YNPTSIDDVVYRFKDVNSICENNINEFKVKNYEVTVLPIYMQIANLHLLLLRDGMIYGDAWNLYRELGFSDQDSFYNHVLDKTKFYINDCLNYYNTGLSNLKLDPNNSW
IDITRYCRFMTFYILDMISICPIYDTKVYDKPINMQTLTRKVYSDPVNFIDENIPISEYEKMYNISPELFSTLFSISFYTNKSGNKFLNGHVNRHVGTDLNYNGLRETH
YGNYGSNYEVESMAFDDIKAYSNNYFNNTQNNNPTSVKSIKFLITKNNDEWIYGEPDSSNIDFTRNIQGYLSNLNNESYTHSLSDMILANNDKIQINIDTPHSYSYSWI
YKGIEDTNYISDKLINQIPLVKEVKLKSRHYSEISVIKGPGFTGGDLILSKVHKPANQIPAQYMKNKITIPIKTKFPAGSQDFKVRLCYASNHDIGLIRLIAGSKYITT
NIQQTFNTTENNPSLIYDDFKYFNFNETLSITSSGIDELYLEFYYSYTDGNFEDFPKLSIPYTRNYSC

>**Cry17Aa_618**

MNNKKIEQNKIVEYNSNLDIQPRELNTLNGLVFTGATVSIILPLIGTTAVVPVVGGVIGIIAALLPVIWPAGTSSNDNLFDAVMKDTEMIMDEKISEYVVNDAMTRLES
LYNILDYYRLSKDFWEKNKDDPLAIAELKERFSKLHSQFIESMAYFKRANYEVLLLPAYANAANLHLLLLREGLLLNKVIDNFITEGLHYEEFKTKRSTYIAHCSTWYN
KGLENIKNKTRDFNKINKYDAYMNLSVLDIISLFLSYDPYQYDKATKLQTLTRTVFSDPLQRAPRDLYISPKEETLFKNLKGLRAFFAEGDLVLTGFRNYFRNTYINDQ
IIEGDLFGYTTNNERYKLFTDSKIYKVTVFIDNVALAIVKLIFHDTDNKEWDFSKTDITDINKYRKEEVYLNLLSNNEIQKEPSHYLYKMHHYGDNYNDSYLFQWIHQS
ISPENYLFDKDKDDNYIITQIPAIKASELSNLGELSLQAIKGPRFTGGNVILSSVSKIDNNDPLYGGTIKIPLLTAFNNTSKFKIRIYYAANHNYNHDYIGALLTINSQ
HVANFKFKQTFSGEDYSNLSYNNYQFDYLVQTVAFPQNTSDVTLNLQFFYDPKFLNDYKQIVIIDKIEFIPEN

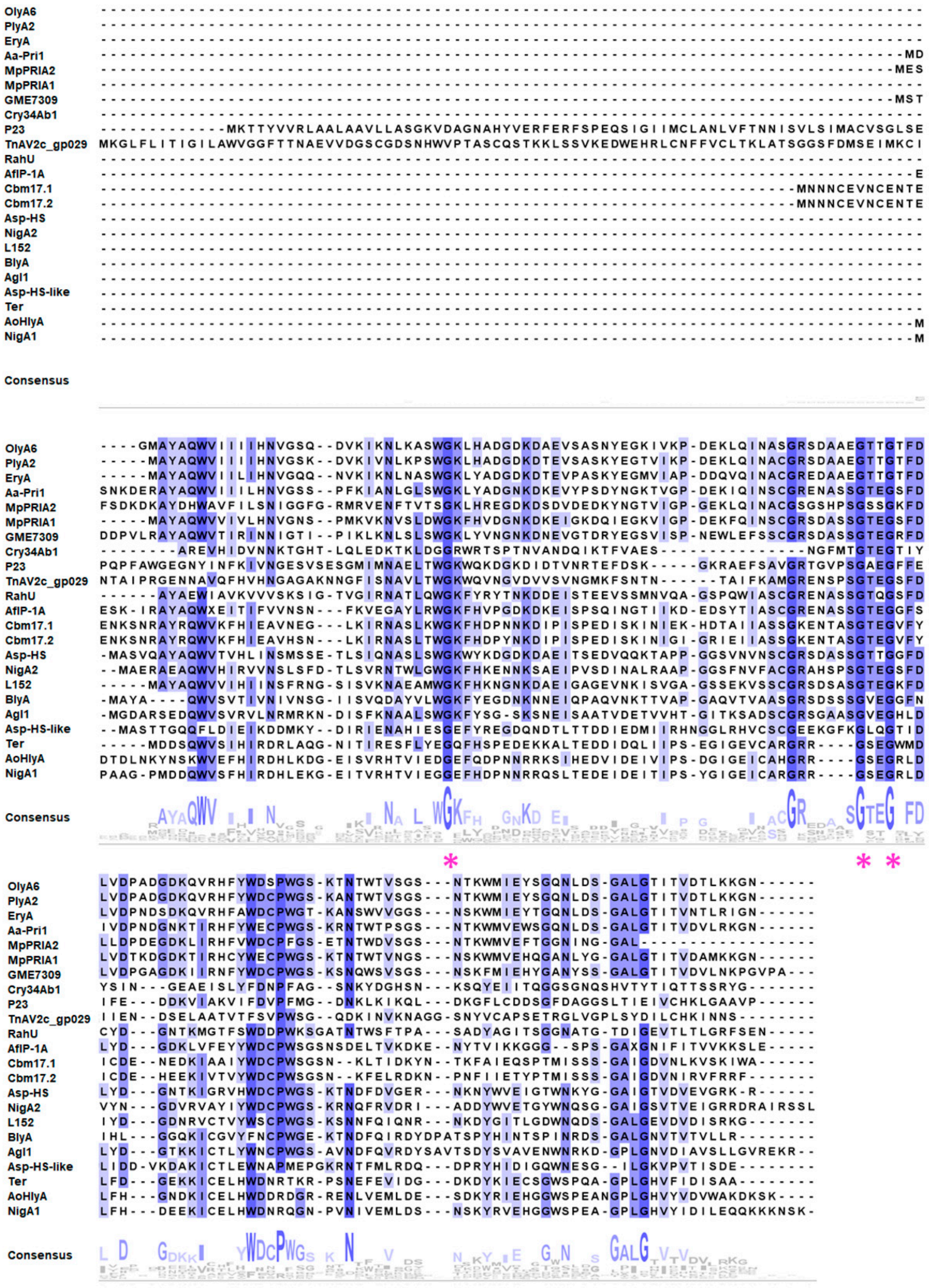

Figure S1. Alignments of aegerolysin amino acid **s**equences. Sequences follow the same order as in the phylogenetic tree (**Figure 2). T**he alignment is stained for percent identity (in blue), consensus sequence is shown below; star, conserved glycine (in magenta). A multiple sequence alignment was performed using the Clustal W/Clustal X algorithm and presented by Jalview [200,201]. OlyA6/PlyA, ostreolysin A6/pleurotolysin A from *Pleurotus ostreatus*, PDB ID: 6MYJ; EryA, erylysin A, and PlyA2, pleurotolysin A2 from *Pleurotus eryngii*; Aa-Pri1, aegerolysin Aa-Pri1 from *Cyclocybe aegerita* (*Agrocybe aegerita*); MpPRIA1, and MpPRIA2, putative aegerolysin genes from *Moniliophthora perniciosa*; GME7309, *Lignosus rhinocerotis* aegerolsin-domain-containing protein; Asp-HS, Asp-hemolysin, and Asp-HS-like, Asp hemolysin-like from *Neosartorya fumigata* (*Aspergillus fumigatus*); Ter, terrelysin from *A. terreus*; AoHlyA, *A. oryzae* hemolysin; NigA1 and NigA2, nigerolysin A1 and A2 from *A. niger*; BlyA, beauveriolysin A from *Beauveria bassiana*; Agl1, *Hypocrea atroviridis* (*Trichoderma atroviride*) aegerolysin; L152, *Alternaria geisen* aegerolysin; Cry34Ab1 (Gpp34Ab1) 13.6 kDa insecticidal crystal protein PDB ID: 4JOX from *Bacillus thuringiensis*; Cbm17.1 and Cbm17.2, hemolysin-like protein Cbm17.1 and Cbm17.2 from *Paraclostridium bifermentans* (*Clostridium bifermentans*); AfIP-1A, two-component insecticidal protein 16 kDa unit, PDB ID: 5V3S, from *Alcaligenes faecalis*; RahU, RahU protein, PDB ID: 6ZC1, from *Pseudomonas aeruginosa*; P23, protein 23 from *Chrysodeixis includes* (*Pseudoplusia includes*); and TnAV6a1 gp029; ORF029 from *Trichoplusia ni* ascovirus 6a1 (2c).

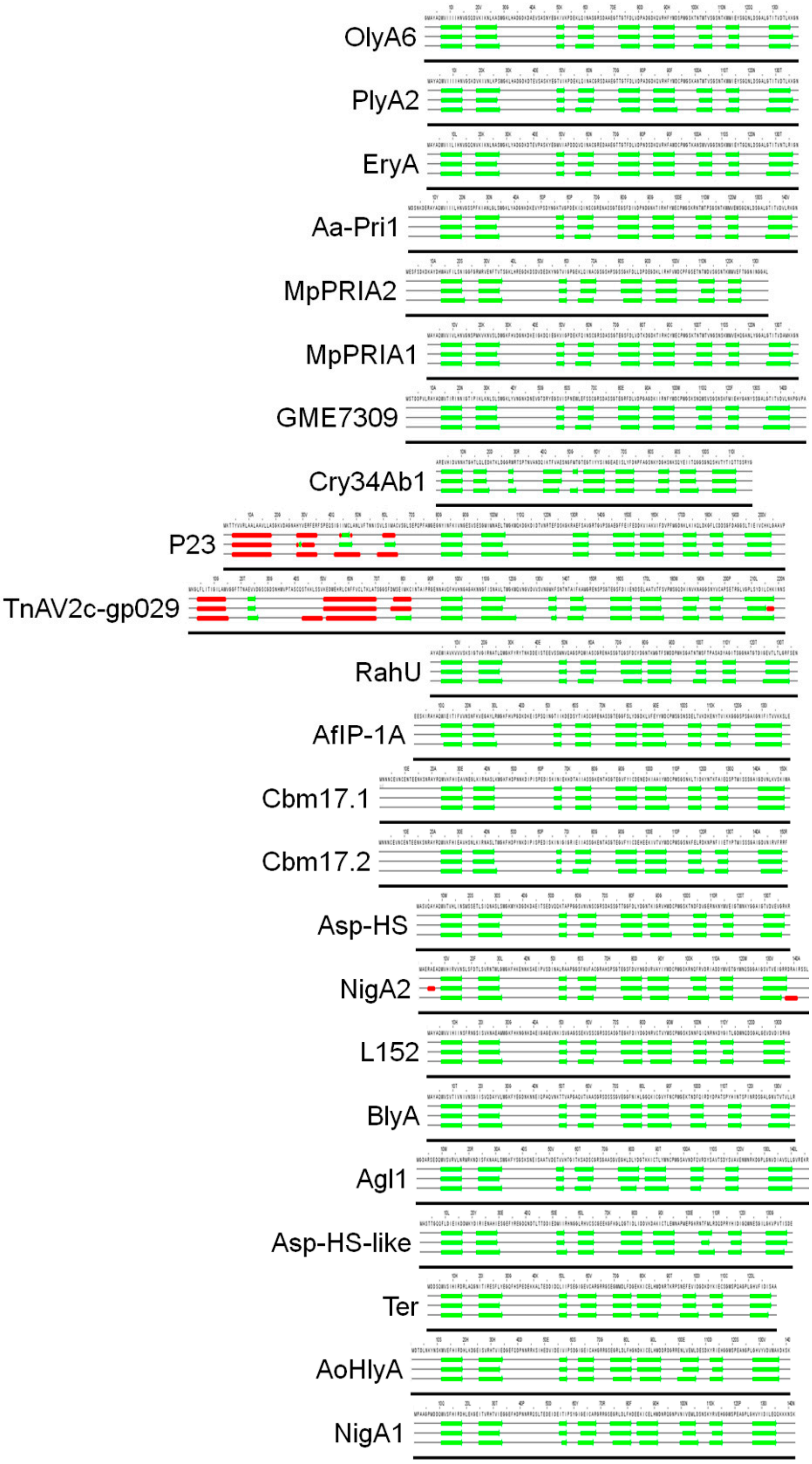

Figure S2. Prediction of secondary structures of aegerolysins. Sequences follow the same order as in the phylogenetic tree (**Figure 2)** and are centered on the first sheet of OlyA6**.** First row: JNetPRED, the consensus prediction; second row: JNetHMM, prediction based on the HMM profile; and third row: JNETPSSM, PSSM-based prediction. Sheets are shown in green and helices in red. Secondary structure of proteins was predicted using the JPred4 server accessible through Jalview [200,202]. OlyA6/PlyA, ostreolysin A6/pleurotolysin A from *Pleurotus ostreatus*, PDB ID: 6MYJ; EryA, erylysin A, and PlyA2, pleurotolysin A2 from *Pleurotus eryngii*; Aa-Pri1, aegerolysin Aa-Pri1 from *Cyclocybe aegerita* (*Agrocybe aegerita*); MpPRIA1, and MpPRIA2, putative aegerolysin genes from *Moniliophthora perniciosa*; GME7309, *Lignosus rhinocerotis* aegerolsin-domain-containing protein; Asp-HS, Asp-hemolysin, and Asp-HS-like, Asp hemolysin-like from *Neosartorya fumigata* (*Aspergillus fumigatus*); Ter, terrelysin from *A. terreus*; AoHlyA, *A. oryzae* hemolysin; NigA1 and NigA2, nigerolysin A1 and A2 from *A. niger*; BlyA, beauveriolysin A from *Beauveria bassiana*; Agl1, *Hypocrea atroviridis* (*Trichoderma atroviride*) aegerolysin; L152, *Alternaria geisen* aegerolysin; Cry34Ab1 (Gpp34Ab1) 13.6 kDa insecticidal crystal protein PDB ID: 4JOX from *Bacillus thuringiensis*; Cbm17.1 and Cbm17.2, hemolysin-like protein Cbm17.1 and Cbm17.2 from *Paraclostridium bifermentans* (*Clostridium bifermentans*); AfIP-1A, two-component insecticidal protein 16 kDa unit, PDB ID: 5V3S, from *Alcaligenes faecalis*; RahU, RahU protein, PDB ID: 6ZC1, from *Pseudomonas aeruginosa*; P23, protein 23 from *Chrysodeixis includes* (*Pseudoplusia includes*); and TnAV6a1 gp029; ORF029 from *Trichoplusia ni* ascovirus 6a1 (2c).
